# Mendelian randomization evidence based on European ancestry for the causal effects of leukocyte telomere length on prostate cancer

**DOI:** 10.1186/s40246-024-00622-8

**Published:** 2024-06-03

**Authors:** Xinrui Wu, Cong Hu, Tianyang Wu, Xinxing Du, Zehong Peng, Wei Xue, Yonghui Chen, Liang Dong

**Affiliations:** Department of Urology, Renji Hospital, Shanghai Jiao Tong University School of Medicine, 160 Pujian Road, Shanghai, 200127 China

## Abstract

**Background:**

Several lines of evidence suggest that leukocyte telomere length (LTL) can affect the development of prostate cancer (PC).

**Methods:**

Here, we employed single nucleoside polymorphisms (SNPs) as instrumental variables (IVs) for LTL (n = 472,174) and conducted Mendelian randomization analysis to estimate their causal impact on PCs (79,148 patients/61,106 controls and 6311 patients/88,902 controls).

**Results:**

Every 1-s.d extension of LTL increased the risk of PCs by 34%. Additionally, the analysis of candidate mediators between LTL and PCs via two-step Mendelian randomization revealed that among the 23 candidates, Alzheimer’s disease, liver iron content, sex hormone binding global levels, naive CD4–CD8-T cell% T cell, and circulating leptin levels played substantial mediating roles. There is no robust evidence to support the reverse causal relationship between LTL and the selected mediators of PCs. Adjusting for the former four mediators, rather than adjusting for circulating leptin levels, decreased the impact of LTL on PCs.

**Conclusion:**

This study provides potential intervention measures for preventing LTL-induced PCs.

**Supplementary Information:**

The online version contains supplementary material available at 10.1186/s40246-024-00622-8.

## Introduction

Globally, prostate cancer (PC) remains the second most common cancer among men [1]. Telomeres are a pair of cap-like structures located at the two ends of each chromosome [2], and are indispensable for cell proliferation and ensuring the replication ability of cells [3]. Recent studies have preliminarily identified the complex genetic correlation between telomere length (TL) and PCs, indicating that longer TL in the stroma and epithelium is negatively correlated with the occurrence of PCs [4–6]. Mechanistically, shorter TL in the these cells may elicit a DNA damage response and activate a senescence-associated secretory pathway that is characterized by increased production of pro-inflammatory cytokines and matrix-degrading proteases [6, 7], resulting in accelerated tumor progression. However, a series of studies found that high incidence rate and mortality of PC are often associated with longer leukocyte telomere length(LTL), impacted comprehensively by genetic, lifestyle, and environmental factors [8–12]. Mechanistically, a longer TL in circulating leukocytes is associated with enhanced leukocyte activities [13]. It is suggested that groups of immunosuppressive leukocytes are more potently activated, resulting in immune evasion and increased risk of PC [14, 15].

The Mendelian randomization (MR), as the name suggests, involves a genetic study design that employs the “randomization” of genetic traits at birth to explore a potential causal relationship between a genetically determined factor and an outcome [16]. Compared with traditional epidemiological studies that are often affected by confounders or by inversive causal associations, MR involves alleles that follow the Mendelian-independent allocation law, which renders the estimated effect more representative of the actual situation [17]. In addition, the time needed to perform MR studies is much shorter than do traditional randomized controlled trials (RCTs), which enables timely updates of issues of concern with the MR tool. To identify potential mechanisms and explore whether mediators exert their mediating effect, two-step MR may be employed [18]. To take a step further, Multivariate Mendelian Randomization (MVMR), which is based on univariate Mendelian Randomization (UVMR), randomly groups multiple variables at the same time, establishes a random distribution of variables between each group, and explores the independent impact of exposure on the results [19].

Previous UVMR studies revealed that a longer genetically determined LTL was associated with a greater risk of PCs [20, 21]. A growing amount of epidemiological evidence advocates that adjusting for the physiological indices, nutrition intake, habits and customs, improving immunity, preventing viral infection, and treating several diseases to regulate modifiable metabolic risk factors have potential benefits for preventing PCs [22–26]. To date, the roles of the potential modifiable risk factors in the pathway through which LTL leads to PCs have not been determined. Exploring this topic with MR methods may help to deepen our understanding of the etiology of PCs, and provide information for improving strategies to prevent and intervene in PCs.

In this MR study, we investigated the independent causal impact of LTL on PCs and evaluated the mediating effects of 23 targetable candidate mediators in the association pathway to identify novel strategies for the prevention and intervention of PCs.

## Materials and methods

### Study design

**Fig. 1:**
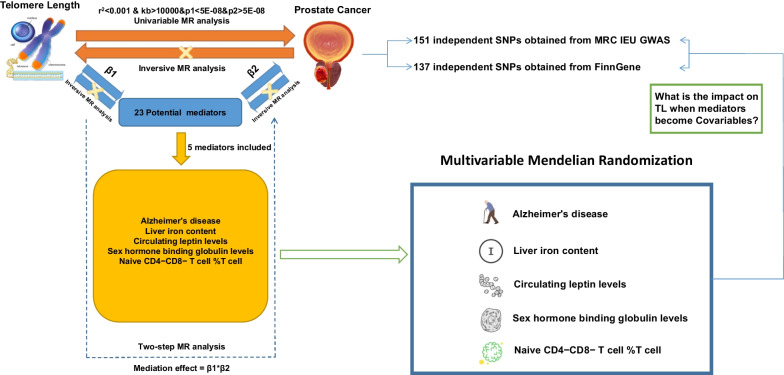
Overview of the MR study design. MR, Mendelian randomization; TL, telomere length; PCs, prostate cancers; SNP, Single Nucleotide Polymorphism; GWAS, genome-wide association studies

This MR study consisted of 3 parts (Fig. 1). Firstly, we used GWAS to conduct UVMR analysis of two sample groups to evaluate the causal impact of LTL on the risk of developing PCs. In the second step, 25 candidate mediators in the pathway between LTL and PCs were screened, and two-step MR was subsequently applied to evaluate the mediating role of each selected mediator in the causal relationship between LTL and PCs. Ultimately, multivariate MR was performed to further analyze the effect of the mediators on PCs and estimate the independent impact of LTL on PCs with adjustment for traits of 5 selected mediators. Notably, considering that PCs occur only in male patients, we excluded SNPs located on the X and Y chromosomes.

### Selection of data sources and adherence to the basic assumptions of MR

The sources of the genetic instruments employed in this study are shown in Table S1. First, we screened SNPs with genome-wide significance (*P* < 5e−08) to satisfy the correlation hypothesis (IVs were closely related to exposure). Second, we set the linkage disequilibrium threshold of r^2^ < 0.001 within a 10,000 kb window and removed SNPs for incompatible alleles and for those palindromic with intermediate allele frequencies to harmonize the impact of SNPs on exposure and outcome to select independent genetic variants to satisfy the independence hypothesis (IVs were not associated with confounders). Third, to satisfy the exclusivity hypothesis, we ensured that the SNPs of the instrumental variables (IVs) we selected were not related to the outcome (*P* < 5e−08) [27].

#### Leukocyte telomere length

Summary statistics for genetic variants associated with their own LTL were extracted from the MRC IEU GWAS of 472,174 European individuals from the UK Biobank [28]. After removing unqualified samples, the authors reported the creation, quality assurance, and initial interference of LTL measurements in the DNA samples of 474,074 participants from 489,090 received DNA samples via a Real-time Quantitative PCR (qPCR) detecting system. Specifically, LTL was measured by the ratio of telomere repeat copy number (T) to the number of single-copy gene (S, HBB, encoding the human hemoglobin subunit β). The T/S ratio for each well, as well as the average T/S and the c.v. for the sample replicates were calculated. Then, strict, predefined quality control (QC) standards at the sample and run levels were applied, and valid measurement results were accepted. All samples that did not meet the QC standards were retested until valid measurement results were obtained, or the samples were considered to be noncompliant or exhausted. Additionally, We collected 3 GWAS data that did not overlap with the UK Biobank for validation of the conclusions [29–31].

#### Candidate mediators

Based on previously published research, we screened 23 candidate mediators, which were classified by category into physical index, nutrition, immunity, viral infection, habits and customs, and disease, based on 3 main criteria. Firstly, based on common scientific knowledge, candidate mediators may be located on the path from LTL to PCs. Secondly, candidate mediators may be altered via lifestyle adjustments or available clinical interventions. Thirdly, the GWAS data of candidate mediators should be applicable to individuals of European or major European ancestry. The potential mediators we selected for further analysis and reasons for the selection, which are mainly based on previous research, are summarized in Table S15. The physical indices included liver iron content [32, 33], body fat percentage [34], sex hormone binding globulin levels [35, 36], circulating leptin levels [37, 38], mean corpuscular hemoglobin [39], and arm fat percentage (left and right) [40]. The nutritional component contains total fatty acids [41], polyunsaturated fatty acids [42], saturated fatty acids [43], vitamin D [44], vitamin E [45], and fresh tomato intake [46]. Naive CD4–CD8-T cell %T cell are involved in immunity [47]. The virus infection part was mainly represented by Epstein Barr virus antibody levels [48, 49]. We included 5 habits and customers related to PCs, including coffee intake [50], alcoholic drinks per week [51], lifetime number of sexual partners [52], cigarettes smoked per day [53], and fried potatoes intake [54]. Three potential diseases associated with PCs were hypertension [55, 56], Alzheimer’s disease [57], and coronary heart disease [58].

The screening criteria of mediators for the causal relationship between LTL and PCs are as follows: (1) LTL should have a causal relationship with each mediator, but the inversive relationship must be absent; (2) mediators should have a causal relationship with PCs; and (3) the relationship between LTL and mediators and the relationship between mediators and PCs should be in the same orientation.

#### Prostate *cancer*

The data of PC patients were obtained from the PRACTICAL and FinnGene Consortium. The data from the PRACTICAL Consortium extracted genetic associations with PCs from GWAS analysis of more than 140,000 males, including 79,148 cases of PCs and 61,106 controls of European ancestry [59]. The data for the other PCs were derived from FinnGene, which included 6311 PC patients and 88,902 controls of European ancestry. FinnGen integrates imputed genotype data generated from new and legacy samples collected by the Finnish Biobank with the Finnish health registry (https://www.finngen.fi/en), which utilities data from the nationwide health register collected since 1969 from every resident in Finland. There was no sample overlap between the two consortia [60].

### Statistical analysis

#### UVMR analyses

We performed the inversive variance weighted (IVW) method as the main analysis, using MR Egger, weighted median, simple mode and weighted mode methods to evaluate the robustness of IVW estimation under other assumptions. Specifically, the IVW method is a causal estimation method that uses random effects to perform a meta-analysis of Wald ratios for multiple site effects in MR analysis of multiple SNPs [61]. The MR Egger method, as a precision-limiting method, does not force the regression line to pass through the origin and allows for targeted gene pleiotropy in the included IVs, and was employed to identify and adjust for potential pleiotropy bias [62]. The weighted median method is the median of the distribution function obtained by ranking the effect values of all individuals’ SNPs according to their weights. When at least 50% of the information comes from effective IVs, the weighted median method can obtain robust estimates [63]. The simple mode and weighted mode methods accumulate SNPs on the basis of the similarity of causal effects and estimate causal effects according to the largest cluster of SNPs [64].

#### Mediator MR analysis

We conducted two-step MR to evaluate whether each mediator had a mediating effect on the relationship between LTL and PCs. The first step was to use UVMR to evaluate the causal impact of LTL on each mediator (β_1_). Inversive MR was conducted between each mediator and LTL to determine whether each mediator interferes with LTL in reverse and consequently affects the effectiveness of the mediation model. In step 2, we performed UVMR to estimate the causal impact of each mediator on PCs (β_2_). Furthermore, inversive MR was employed between PCs and each mediator to analyze whether there was a reverse causal relationship between them. The proportions of mediation associated between LTL and PCs were calculated as β_1_ × β_2_/β_Total_ [65], where β_Total_ was the evaluation of the causal impact of LTL on PCs via UVMR. In addition, the delta method was used to obtain the standard error (SE) of β_1_ × β_2_ and calculate the 95% confidence interval (95% CI) of the mediation proportions [66].

#### MVMR analysis

MVMR was conducted to evaluate the direct impact of LTL and each mediator on PCs, with adjustments to each other to determine the effect of each mediator on LTL and PCs. MVMR analysis conformed to 3 critical assumptions: (1) genetic variation must be closely related to exposure in UVMR analysis, and must be vigorously related to at least one of the multiple exposures in MVMR analysis; (2) genetic variation was not associated with confounding factors related to the association between the instruments of each exposure and PCs; (3) the impacts of genetic variation on PCs must go through each exposure [67]. In MVMR, we employed the MV-IVW method as the main analysis method, which yields the most accurate and unbiased causal estimation [68].

All analyses in this study were conducted via R packages TwoSampleMR (version 0.5.7), robustbase (version 0.99–0), MVMR (version 0.3), MRPRESSO (version 1.0), MendelianRandomization (version 0.8.0) and fdrtool (version 1.2.17) in R software (version 4.3.0). A *P*-value < 0.05 was considered to indicate statistical significance. The results of IVW were considered causal associations only when they had the same direction and statistical significance in at least one sensitivity analysis, without evidence of pleiotropy. We calculated the adjusted q-value via the false discovery rate (FDR) method to correct for the *P*-value. The IVW results, with *P* < 0.05 and FDR q-values < 0.05, were classified as strong evidence and included in subsequent analysis.

#### Heterogeneity and horizontal pleiotropy

We used Cochran’s Q test to assess the heterogeneity of SNPs. A *P*-value of the Q statistic < 0.05 indicated that the included SNPs have heterogeneity [62]. In addition, horizontal pleiotropy was determined based on the regression intercept in the MR Egger regression model. A regression intercept of not zero and a *P*-value for intercept (P_intercept_) < 0.05 suggest the existence of horizontal pleiotropy [69]. The F-statistics was to evaluate weak instrument bias. When the F-statistics was < 10, we usually assumed that the genetic variation used was a weak IV, which may introduce bias to the results [70].

## Results

### Basic process framework of this MR study

Figure 1 displays the basic process framework of the MR research. Information on the GWAS datasets for case definition and exclusion criteria for included LTL, mediators, and PCs in this MR study is listed in Table S2.

### UVMR analysis of the effects of LTL on PCs

**Fig. 2:**
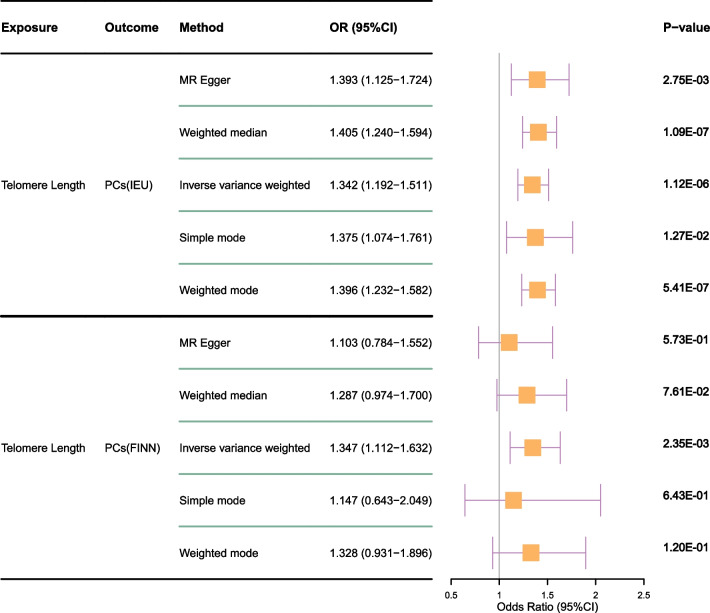
Two sample MR analysis of the genetically causal relationship between LTL and PCs derived from GWAS data from PRACTICAL and FinnGene, respectively. MR, Mendelian randomization; TL, telomere length; PCs, prostate cancers; GWAS, genome-wide association studies

PRACTICAL and FinnGen exhibited high consistency in terms of UVMR results for PCs (Fig. 2). The IVW results of the PRACTICAL consortium suggested that each genetically predicted 1-s.d longer LTL was associated with higher PC risk (odds ration [OR]: 1.342; [95% CI 1.192–1.1511]; *P* = 1.12E−06), and the analysis of validation consortium FinnGen also supported this result (OR: 1.347; [95% CI 1.112–1.632]; *P* = 2.35E−03). Additionally, results of external GWAS data in both consortium were also solid(OR: 1.38; [95% CI 1.14–1.69]; *P* = 1.22E−03; OR: 1.37; [95% CI 1.06–1.77]; *P* = 1.44E−02). The UVMR estimates were validated via sensitivity analysis (Table S3). Instrumental validity test indicated sufficient instrumental strength because all F-statistics were > 10 despite heterogeneity among IVs. Additionally, no horizontal pleiotropy was detected (*P* > 0.05; Table S4). The inversive MR results revealed that there was no genetically determined causal relationship between PCs and LTL in either PRACTICAL or FinnGen consortium (Table S5).

### Bidirectional UVMR estimates for the causal effect of LTL on potential mediators

**Fig. 3:**
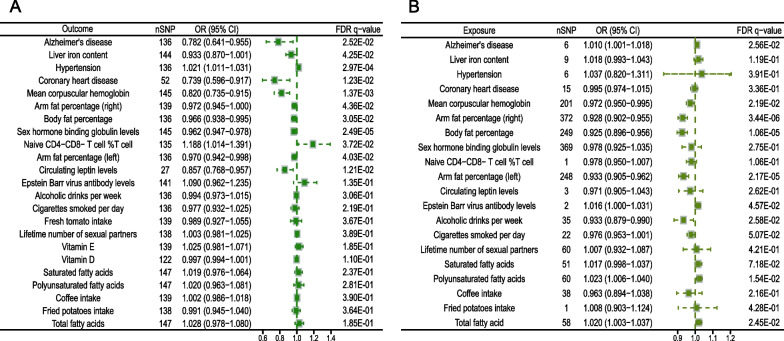
IVW results of Bidirectional univariate MR analysis of LTL on 23 candidate mediators. **A** LTL data from MRC IEU GWAS is used as exposure to analyze the impact of LTL on 23 candidate mediators; **B** Exploration of the effects of 23 candidate mediators on LTL based on LTL from MRC IEU GWAS. MR, mendelian randomization; IVW, inverse-variance weighted; LTL, lymphocyte telomere length; OR, odds Ratio; CI, confidence interval; FDR, false discovery rate; GWAS, genome-wide association studies

Eleven of the 23 candidate mediators had causal relationships with PC risk (Fig. 3A). Causal associations of genetically determined, as solid evidence, suggested significant correlations between each 1-s.d longer LTL and Alzheimer’s disease (OR: 0.782; [95% CI 0.641 − 0.955]; FDR q − value = 2.52E−02), liver iron content (OR: 0.933; [95% CI 0.870 − 1.001]; FDR q − value = 4.25E−02), hypertension (OR: 1.021; [95% CI 1.011 − 1.031]; FDR q − value = 2.97E−04), coronary heart disease (OR: 0.739; [95% CI 0.596 − 0.917]; FDR q − value = 1.23E−02), mean corpuscular hemoglobin (OR: 0.820; [95% CI 0.735 − 0.915]; FDR q − value = 1.37E−03), arm fat percentage (right; OR: 0.972; [95% CI 0.945 − 1.000]; FDR q − value = 4.36E−02), body fat percentage (OR: 0.966; [95% CI 0.938 − 0.995]; FDR q − value = 3.05E−02), sex hormone binding globulin levels (OR: 0.962; [95% CI 0.947 − 0.978]; FDR q − value = 2.49E−05), naive CD4–CD8-T cell %T cell (OR: 1.188; [95% CI 1.014 − 1.391]; FDR q − value = 3.72E−02), arm fat percentage (left; OR: 0.970; [95% CI 0.942 − 0.998]; FDR q − value = 4.03E−02) and circulating leptin levels (OR: 0.857; [95% CI 0.768 − 0.957]; FDR q − value = 1.21E−02) after FDR adjustment for multiple comparisons (Table S6). The mean F-statistics of IVs were all greater than 100, suggesting that the possibility of weak tool bias was limited. Although most heterogeneity of IVs might exist, these results did not show horizontal pleiotropy between LTL and candidate mediators (all P_intercept_ > 0.05; Table S7).

Nine of the 23 candidate mediators have inversive causal relationships with risk of PCs (Fig. 3B). Genetically determined causal relationships indicated that Alzheimer’s disease (OR: 1.018; [95% CI 1.001 − 1.018]; FDR q − value = 2.56E−02), mean corpuscular hemoglobin (OR: 0.972; [95% CI 0.950 − 0.995]; FDR q − value = 2.19E−02), arm fat percentage (right; OR: 0.928; [95% CI 0.902 − 0.955]; FDR q − value = 3.44E−06), body fat percentage (OR: 0.925; [95% CI 0.896 − 0.956]; FDR q − value = 1.06E−05), arm fat percentage (left; OR: 0.933; [95% CI 0.905 − 0.962]; FDR q − value = 2.17E−05), Epstein Barr virus antibody levels (OR: 1.016; [95% CI 1.000 − 1.031]; FDR q − value = 4.57E−02), alcoholic drinks per week (OR: 0.933; [95% CI 0.879 − 0.990]; FDR q − value = 2.58E−02), polyunsaturated fatty acids (OR: 1.023; [95% CI 1.006 − 1.040]; FDR q − value = 1.54E−02), and total fatty acid (OR: 1.020; [95% CI 1.003 − 1.037]; FDR q − value = 2.45E−02) showed prominent association with LTL for every 1-s.d change (Table S8). Similarly, there was heterogeneity between IVs, but with all average F-statistic of IVs > 100, the results were considered reliable. Furthermore, no horizontal pleiotropy was detected between LTL and the candidate mediators (all P_intercepts_ > 0.05; Table S9).

Seven of the 23 candidate mediators met the screening criteria and were included in subsequent MR analysis. In terms of Alzheimer’s disease, although the results of inversive MR indicated a genetically determined impact on LTL, we inferred that this effect was caused by an underlying negative feedback mechanism, and that Alzheimer’s disease is still a potential mediator of LTL-mediated PCs rather than a possible confounding factor [71].

### Bidirectional UVMR estimates for the causal effect of potential mediators on PCs

**Fig. 4:**
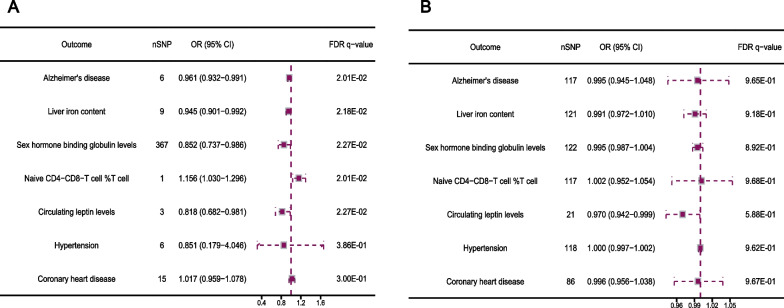
Bidirectional univariate MR analysis of 11 candidate mediators screened form all 23 mediators on PCs. **A** The impact of 11 candidate mediators on PCs, including IVW results for inclusion with nSNP > 1 and Wald Ratio results for inclusion with nSNP = 1; **B** The IVW results of PCs from MRC IEU GWAS affecting on 11 candidate mediators. MR, mendelian randomization; PCs, prostate cancers; IVW, inverse-variance weighted; OR, odds Ratio; CI, confidence interval; FDR, false discovery rate; GWAS, genome-wide association studies

UVMR analysis was used to explore genetically determined causal relationships from each mediator to PCs. Specifically, 1-s.d unit higher Alzheimer’s disease (OR: 0.961; [95% CI 0.932 − 0.991]; FDR q − value = 2.01E−02), liver iron content (OR: 0.945; [95% CI 0.901 − 0.992]; FDR q − value = 2.18E−02), sex hormone binding globulin levels (OR: 0.852; [95% CI 0.737 − 0.986]; FDR q − value = 2.27E−02), and circulating leptin levels (OR: 0.818; [95% CI 0.682 − 0.981]; FDR q − value = 2.27E−02) were related to a decreased risk of PCs, while each 1-s.d unit higher naive CD4–CD8-T cell %T cell (OR: 1.156; [95% CI 1.030 − 1.296]; FDR q − value = 2.01E−02) was related to an increased risk of PCs (Fig. 4A). We did not observe horizontal pleiotropy in the MVMR model. In addition, the F-statistic range of all IVs in the MVMR model was 33.960 to 265.120, which suggests that IVs have sufficient instrument strength despite the existence of heterogeneity (Table S10-11).

In contrast, inversive MR indicated that there was no genetically determined causal relationship among all 7 candidate mediators in PCs after FDR adjustment (Fig. 4B). Similarly, the F-statistics in the MVMR model were greater than 100 for all the models, revealing the robustness of IVs (Table S12). In addition, we also found heterogeneity and the absence of pleiotropy (Table S13).

### Two-step MR estimates to reveal mediating effects of each mediator in the association between LTL and PCs

**Fig. 5:**
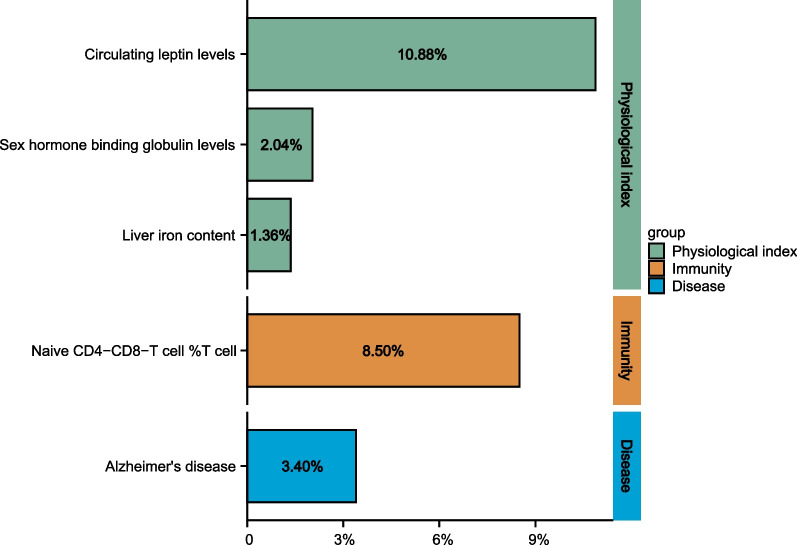
proportion of Two-step MR estimates for the causal influence of LTL on PCs via each mediator. MR, Mendelian randomization; TL, telomere length; PCs, prostate cancers

Three selected mediator categories, namely physical index, immunity, and disease, were ranked based on the proportion of the effect they mediate in the association between LTL and PCs. The physical indices were ranked first, and included circulating leptin levels (10.88%), sex hormone binding global levels (2.04%), and liver iron content (1.36%). It was followed by the immunity mediator, namely naive CD4–CD8-T cell% T cell (8.50%). Disease, namely Alzheimer’s disease (3.40%; Fig. 5), contributed to the smallest proportion.

### MVMR of LTL adjusted by mediators on PCs

**Fig. 6:**
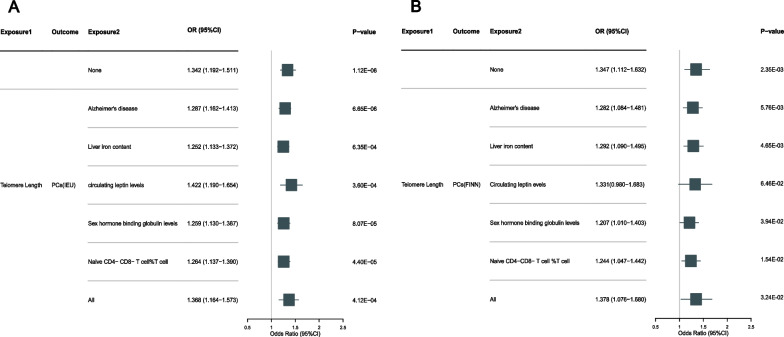
Univariate and multivariable MR estimates for the causal, independent effect of LTL on PCs form two consortiums without overlapping samples. **A**, **B** Based on 79,148 samples from MRC IEU GWAS and 6311 samples in FinnGene, MR estimates were obtained via the IVW method in UVMR and the MV-IVW method in MVMR. The data is represented by OR (95% CI), which means that for every increase in telomere length by 1-SD, the risk of PCs increases by 34.2% in MRC IEU GWAS and 34.7% in FinnGene, respectively. MR, Mendelian randomization; TL, telomere length; PCs, prostate cancers; GWAS, genome-wide association studies; IVW, inverse-variance weighted; UVMR; Univariate mendelian randomization; MVMR, Multivariable mendelian randomization; OR, odds Ratio; CI, confidence interval; SD, Standard Deviation

After adjusting for Alzheimer’s disease (IVW OR: 1.287; [95% CI 1.162 − 1.413]), liver iron content (IVW OR: 1.252; [95% CI 1.133 − 1.372]), sex hormone binding globulin levels (IVW OR: 1.259; [95% CI 1.130 − 1.387]) and naive CD4–CD8-T cell% T cell (OR: 1.264, [95% CI 1.137 − 1.390]), the risk of LTL on PCs was reduced, while adjusting for circulating leptin levels (IVW OR: 1.422; [95% CI 1.190 − 1.654]), did not induce a pronounced effect (Fig. 6A). We subsequently adjusted for all the selected mediators and found an increase in the impact of LTL on PCs (OR: 1.368; [95% CI 1.164 − 1.573]). The results of the validation consortium FinnGene also support this viewpoint (Fig. 6B). In addition, the MVMR-Egger method confirmed the robustness of all estimates of the MVMR-IVW method, with an F-statistic range of 16.936–121.621, indicating a lower risk of bias caused by horizontal pleiotropy, and all directions of the IVW results in MVMR were consistent with at least one sensitivity analysis result (Table S14).

### UVMR estimates of the effects of nutritional factors and habits and customs factors on the five causal mediators of PCs

UVMR estimates revealed significant correlations between an 1-s.d increase in sex hormone binding globulin levels and total fatty acids (OR: 0.740; [95% CI 0.662–0.827]; FDR q − value = 2.37E−06), saturated fatty acids (OR: 0.713; [95% CI 0.641–0.792]; FDR q − value = 2.91E−08), vitamin D (OR: 1.007; [95% CI 1.002–1.012]; FDR q − value = 3.14E−02), and coffee intake (OR: 1.047; [95% CI 1.015–1.080]; FDR q − value = 3.35E−02). In addition, we observed significant correlations between an 1-s.d increase in naive CD4–CD8-T cell %T cell and total fatty acids (OR: 0.840; [95% CI 0.791–0.891]; FDR q − value = 2.90E−07), polyunsaturated fatty acids (OR: 0.840; [95% CI 0.792–0.891]; FDR q − value = 2.56E−07), saturated fatty acids (OR: 0.861; [95% CI 0.811–0.914]; FDR q − value = 1.48E−05), and alcoholic drinks per week (OR: 1.044; [95% CI 1.015–1.073]; FDR q − value = 3.14E−02). Furthermore, we observed significant correlations between an 1-s.d increase in Alzheimer’s disease and total fatty acids (OR: 1.058; [95% CI 1.023–1.094]; FDR q − value = 1.49E−02), and saturated fatty acids (OR: 1.044; [95% CI 1.018–1.070]; FDR q − value = 1.04E−02) (Table S16). Although heterogeneity of IVs might exist, with all average F-statistic of IVs > 10, the results were considered reliable. Most of the results did not show horizontal pleiotropy between nutrition, habits and customs factors, and five causal mediators of PCs (P_intercept_ > 0.05). However, we observed horizontal pleiotropy between cigarettes smoked per day and naive CD4–CD8-T cell %T cell, as well as between sex hormone binding globulin levels and polyunsaturated fatty acids, fresh tomato intake, and lifetime number of sexual partners (P_intercept_ < 0.05; Table S17).

## Discussion

This MR study delved into the causal effects of LTL on the risk of PCs, as well as the mediating effects of common and easy-to-intervene mediators including physiological indices, nutrition ingestion, habits and customs, immune factors, virus infection, and several diseases. The increase in genetically determined LTL by 1-s.d has a causal relationship with a 34% increase in prostate cancer. Furthermore, we screened 23 common mediators, and identified 5 causal mediators linking LTL to PCs, among which genetically determined circulating leptin levels and naive CD4–CD8-T cell% T cell played strong mediating roles, and contributed to over 5% of the total impact of LTL on PCs. After adjusting for the causal mediators, the genetically determined causal relationship between LTL and PC was still present but was significantly mitigated.

Our research findings are consistent with previous MR studies [20, 21], which suggested that longer LTL increases the risk of developing PCs. Mechanistically, a longer LTL is associated with enhanced leukocyte activities in the human body [13]. A subgroup of activated leukocytes, namely the naive CD4–CD8-T cell%T cell, produce interleukin-17 (IL-17), mediate immune evasion, and subsequently increase the risk of PC [14, 15]. An alternate mechanism involves reduced compaction of telomeric chromatin and increased telomere fragility caused by excessively long telomeres [72–74], which may lead to telomeric damage and cell dysfunction in anti-tumor lymphocytes. The MR evidence for the causal relationship between LTL and PC may help to improve our understanding of mechanisms in the development of PC, and to accelerate the discovery of novel therapeutics targeting LTL. We also provided evidence that PC, in reverse, does not possess a genetically determined causal relationship with LTL. As PC, especially localized PC, may not be able to drastically influence the hematopoietic microenvironment where lymphocytes derive, or directly influence the lymphocytes in the blood circulation, the LTL information extracted from circulating lymphocytes may not reflect a PC-induced LTL alteration. However, LTL of infiltrating lymphocytes in primary or metastatic lesions may be influenced.

Notably, we identified and quantified the mediating roles of 5 mediators between LTL and PCs, namely circulating leptin levels, sex hormone binding globulin levels, liver iron content, naïve CD4–CD8-T cell %T cell, and Alzheimer’s disease. Among the 5 identified mediators, circulating leptin levels accounted for 10.88% of the total impact of LTL on PCs. The impact, as well as the negative correlation between LTL and circulating leptin levels were supported by previous research [75]. Furthermore, evidence from both an RCT and an observational study has suggested a negative correlation between circulating leptin levels and PCs [76, 77]. The negative impact of leptin on PC might involve an anti-proliferative and anti-angiogenetic effect of leptin on prostate cancer cells, but the underlying mechanisms remain unclear [78]. The level of sex hormone binding globulin accounted for 2.04% of the total impact of LTL on PCs. Furthermore, a negative correlation between sex hormone binding protein levels and PC risk was revealed in an MR study [79]. Sex hormone binding protein binds androgens with high affinity and regulates its bioavailability [80]. As an androgen-driven cancer, it is plausible that excessive sex hormone binding protein may result in a decreased androgen level and reduced PC risk [81]. An observational study suggested that a high body iron status is associated with a shorter LTL in the American population, especially in adults aged 65 or older [82]. In addition, an MR study revealed the protective role of iron in the development of prostate cancer [83]. The mechanism for how iron status influences the risk of PC remains unknown. A possible hypothesis of the protective role of iron in PC involves ferroptosis, a form of regulated cell death that inhibits some types of cancers including prostate cancer [83, 84]. For every 1-sd increase in LTL, the risk of Alzheimer’s disease decreased by 21.8%. Furthermore, the mechanism underlying the impact of Alzheimer’s disease on the risk of PC is still under investigation, although one hypothesis suggests the dysregulation of the immune system [85]. Specifically, neuroinflammation in Alzheimer’s disease results in activated immune and inflammatory responses in peripheral tissues, and subsequent surveillance and elimination of neoplastic cells [85, 86]. The results supported the mediating role of the three physiological indices and Alzheimer’s disease in the causal effects of LTL on PCs, adding to the immune index naive CD4–CD8-T cell%T cell discussed in the previous paragraph.

After adjusting for 4 of the 5 causal mediators, the impact of LTL on PCs decreased, indicating that the mediators played a promoting role in the process from LTL to PCs. However, adjusting for the circulating leptin levels, another causal mediator, was unable to elicit a similar effect, which may be attributed to residual confounding effects. Specifically, the circulating leptin levels may affect other potential mediators of PCs that were not included in the study, resulting in concealed effects of their own.

Nevertheless, although other physiological indices, including body fat percentage, mean corpuscular hemoglobin, arm fat percentage (right) and arm fat percentage (left) had genetically determined causal effects on both LTL and PCs, inversive MR reflected that they, as confounding factors, would interfere with the causal effect of LTL on PCs. Thus, they did not meet the criteria for eliciting mediating effects.

We did not observe a causal relationship from LTL to nutritional factors, virus infection, or habits and customs. Interestingly, via inversive MR, we found that total fatty acids and polyunsaturated fatty acids had positive impacts on LTL, which is consistent with the findings of other researchers [87, 88]. Notably, for every 1-s.d increase in alcoholic drinks per week, LTL shortened by 6.7%, which was also suggested by an MR study [89].

Compared with previous MR studies, this is the first MR study to identify causal mediators between LTL and PCs and explore their impact on this pathway, independently. The identification of the modifiable risk factors in the pathway through which LTL leads to PCs may offer additional options for the prevention of PCs. The following are the main advantages of this study. Firstly, to ensure the repeatability and effectiveness of the results based on PRACTICAL and to maximize statistical capabilities, we employed 2 almost non overlapping GWAS sources for PCs and 3 non overlapping GWAS sources for LTL to validate our findings. In addition, multiple MR sensitivity analyses were performed to examine the robustness of IVW results, each of which excluded interference from horizontal pleiotropy. Furthermore, reverse MR analyses were conducted between LTL, mediators, and PCs to reduce the reverse causal relationship between mediators and LTL, and to analyze the comprehensive interrelationships among the 3 entities. Several limitations exist in this study. First, due to the different consortiums from which the IVs were derived from, the persistence of heterogeneity in IVs may still affect the robustness of our MR results via potential biases despite the employment of random effects models [90]. Second, we cannot explicitly explain the mediating effects between LTL and PCs in this study, despite strict screening of candidate mediators. For instance, several potential non genetic candidate mediators, such as environment and occupation, were not available in GWAS [91]. Third, the two-step, two sample MR cannot effectively exclude potential interactions between LTL and mediators. Representing IVs with SNPs can greatly alleviate potential biases caused by interactions between LTL and mediators, which may be the reason for the increased risk of LTL on PCs after adjusting for circulating leptin levels. Fourth, this study was mainly based on GWAS conducted among individuals of European descent from high-income countries and cannot represent individuals from other ethnic groups or from low-income countries.

Importantly, LTL remains an independent risk factor for PCs, suggesting that the impact of downstream mediators, including circulating leptin levels, sex hormone binding globulin levels, liver iron content, naïve CD4–CD8-T cell %T cell, and Alzheimer’s disease, on LTL is limited. The results of this study may provide novel ideas for the prevention of PCs by targeting aberrant changes in LTL or by taking precautions against the suggested mediating factors of LTL-induced PCs.

### Supplementary Information

Supplementary Material 1

Supplementary Material 2

## Data Availability

All data is provided in the manuscript and in the supplementary tables.

## Abbreviations

PC: Prostate cancer
LTL: Leukocyte telomere length
IVs: Instrumental variables
UVMR: Univariate Mendelian randomization
MVMR: Multivariate Mendelian randomization
GWAS: Genome-wide association studies
SNP: Single nucleoside polymorphism
qPCR: Quantitative PCR detecting system
IVW: Inversive variance weighted
FDR: False discovery rate
SE: Standard error
OR: Odds ratio
CI: Confidence interval
