# Supplementary material for: Mendelian randomization evidence based on European ancestry for the causal effects of leukocyte telomere length on prostate cancer
Source: Hum Genomics. 2024 Jun 3;18:56. doi: 10.1186/s40246-024-00622-8 (PMC11145789; doi:10.1186/s40246-024-00622-8)
Supplement: Supplementary file 2 — Supplementary Material 2 [file 40246_2024_622_MOESM2_ESM.docx]

**Supplementary material**

**Table of contents**

| **Table S2.** Case definition and exclusion criteria in included TL, Mediators and PCs | ……………………2 |
| --- | --- |
| **Table S3.** MR results for the causal effect of TL on PCs | ……………………5 |
| **Table S4.** Univariable MR pleiotropy and heterogeneity test for the association between TL and PCs | ……………………6 |
| **Table S5.** Inversive MR for the interactive relationships between PCs and TL | ……………………7 |
| **Table S6.** Univariable MR estimates for the causal effect of TL on potential mediators | ……………………8 |
| **Table S7.** Univariable MR pleiotropy and heterogeneity test for the associations between TL and potential mediators | ……………………13 |
| **Table S8.** Inversive MR for the interactive relationships between TL and potential mediators | ……………………15 |
| **Table S9.** Inversive MR pleiotropy and heterogeneity test for the associations between TL and potential mediators | ……………………19 |
| **Table S10.** Univariable MR estimates for the causal effect of potential mediators on PCs | ……………………21 |
| **Table S11.** Univariable MR pleiotropy and heterogeneity test for the associations between potential mediators and PCs | ……………………23 |
| **Table S12.** Inversive MR for the interactive relationships between potential mediators and PCs | ……………………24 |
| **Table S13.** Inversive MR pleiotropy and heterogeneity test for the associations between potential mediators and PCs | ……………………26 |
| **Table S14.** Multivariable MR estimates for the independent effect of TL on PCs with adjustment for other related factors | ……………………27 |
| **Table S15.** Selected mediators of the impact of LTL on PCs and the rationale for selection based on published literature | ……………………37 |
| **Table S16.** Univariable MR estimates for the causal effect between nutrition factors and five causal mediators of PCs | ……………………39 |
| **Table S17.** Univariable MR pleiotropy and heterogeneity test for the associations between nutrition, habits and customs factors with five causal mediators of PCs | ……………………43 |

**Table S2. Case definition and exclusion criteria in included TL, Mediators and PCs**

| **Phenotype** | **Consortium/Cohort** | **Sample size or case/Control** | **Ancestry** | **Year of**  **publication** | **Consortium/Cohort ID** | **PMID** | **Unit** |
| --- | --- | --- | --- | --- | --- | --- | --- |
| TL | MRC IEU GWAS | 472,174 | European | 2021 | ieu-b-4879 | 37117760 | SD |
| TL(Validation) | GWAS | 37,684 |  | 2013 | NA | 23535734 |  |
| TL(Validation) |  | 2,226 |  | 2012 |  | 23001564 |  |
| TL(Validation) |  | 3,554 |  | 2011 |  | 21573004 |  |
| **Mediator: Physiological index** | | | | | | | |
| Liver_iron_content | GWAS Catalog | 32,858 | European | 2021 | ebi-a-GCST90016674 | 34128465 | SD (mg/g) |
| Body_fat_percentage | UK Biobank | 331,117 | European | 2017 | ukb-a-264 | 26833246 | SD |
| Sex_hormone_binding_globulin_levels | GWAS Catalog | 370,125 | European | 2020 | ebi-a-GCST90012111 | 32042192 | SD |
| Circulating_leptin_levels | GWAS Catalog | 49,909 | European | 2020 | ebi-a-GCST90007310 | 32917775 | SD |
| Mean_corpuscular_hemoglobin | GWAS Catalog | 172,332 | European | 2016 | ebi-a-GCST004630 | 27863252 | SD |
| Arm_fat_percentage (right) | UK Biobank | 454,789 | European | 2018 | ukb-b-12854 | 28388634 | SD |
| Arm fat percentage (left) | UK Biobank | 331,198 | European | 2017 | ukb-a-286 | 28388634 | SD |
| **Mediator: Nutrition** | | | | | | | |
| Total_fatty_acids | GWAS Catalog | 114,999 | European | 2020 | met-d-Total_FA | 32114887 | SD |
| Polyunsaturated_fatty_acids | GWAS Catalog | 114,999 | European | 2020 | met-d-PUFA | 32114887 | SD |
| Saturated_fatty_acids | GWAS Catalog | 114,999 | European | 2020 | met-d-SFA | 32114887 | SD |
| Vitamin_D | UK Biobank | 460,351 | European | 2018 | ukb-b-12648 | 29343764 | SD |
| Vitamin_E | UK Biobank | 64,979 | European | 2018 | ukb-b-6888 | 21729881 | SD |
| Fresh_tomato_intake | UK Biobank | 64,949 | European | 2018 | ukb-b-730 | 26861389 | SD |
| **Mediator: Immunity** | | | | | | | |
| Naive_CD4-CD8-_T_cell %T_cell | GWAS Catalog | 3,427 | European | 2020 | ebi-a-GCST90001568 | 32929287 | SD |
| **Mediator: Virus infection** | | | | | | | |
| Epstein_Barr_virus_antibody_levels | GWAS Catalog | 8,191 | European | 2020 | ebi-a-GCST90006901 | 33204752 | SD |
| **Mediator: Habits and customs** | | | | | | | |
| Coffee_intake | UK Biobank | 428,860 | European | 2018 | ukb-b-5237 | 25288136 | SD |
| Alcoholic_drinks_per_week | MRC IEU GWAS | 335,394 | European | 2019 | ieu-b-73 | 30643251 | SD |
| Lifetime_number_of_sexual_partners | UK Biobank | 378,882 | European | 2018 | ukb-b-4256 | 30643258 | SD |
| Cigarettes_smoked_per_day | MRC IEU GWAS | 249,752 | European | 2019 | ieu-b-142 | 30643251 | SD |
| Fried_potatoes_intake | UK Biobank | 64,949 | European | 2018 | ukb-b-12836 | 25994509 | SD |
| **Mediator: Disease** | | | | | | | |
| Hypertension | UK Biobank | 66,341/228,609 | European | 2017 | ukb-a-207 | 28135244 | Event |
| Alzheimer's_disease | FinnGene | 5,918/111,471 | European | 2021 | finn-b-G6_AD_WIDE_EXMORE | NA | Event |
| Coronary_heart_disease | GWAS Catalog | 22,233/64,762 | European | 2011 | ebi-a-GCST000998 | 21378990 | logOR |
| **Outcome: PCs** | | | | | | | |
| PCs | PRACTICAL | 79,148/61,106 | European | 2018 | ieu-b-85 | 29892016 | Event |
| PCs | FinnGene | 6,311/88,902 | European | 2021 | finn-b-C3_PROSTATE | NA | Event |

Abbreviations: TL=Telomere Length; BMR=Basal metabolic rate; PCs=Prostate cancers.

**Table S3. MR results for the causal effect of TL on PCs**

| **LTL data source** | **PCs data source** | **Method** | **No. of SNPs** | **^†^β (95% CI)** | **P value** | **F-statistic** |
| --- | --- | --- | --- | --- | --- | --- |
| MRC IEU GWAS | PCs(IEU) | MR Egger | 151 | 1.39 (1.13-1.72) | 2.75E-03 | 115.99 |
|  |  | Weighted median |  | 1.41 (1.25-1.58) | 1.09E-07 |  |
|  |  | IVW |  | 1.34 (1.19-1.51) | 1.12E-06 |  |
|  |  | Simple mode |  | 1.38 (1.08-1.75) | 1.27E-02 |  |
|  |  | Weighted mode |  | 1.40 (1.22-1.59) | 5.41E-07 |  |
|  | PCs (FinnGene) | MR Egger | 137 | 1.10 (0.78-1.55) | 5.73E-01 | 119.25 |
|  |  | Weighted median |  | 1.29 (0.97-1.70) | 7.61E-02 |  |
|  |  | IVW |  | 1.35 (1.11-1.63) | 2.35E-03 |  |
|  |  | Simple mode |  | 1.15 (0.64-2.05) | 6.43E-01 |  |
|  |  | Weighted mode |  | 1.33 (0.93-1.90) | 1.20E-01 |  |
| GWAS(Validation) | PCs(IEU) | MR Egger | 10 | 1.27 (0.65-2.46) | 5.04E-01 | 48.66 |
|  |  | Weighted median |  | 1.34 (1.16-1.55) | 5.10E-05 |  |
|  |  | IVW |  | 1.38 (1.14-1.69) | 1.22E-03 |  |
|  |  | Simple mode |  | 1.41 (1.13-1.75) | 1.31E-02 |  |
|  |  | Weighted mode |  | 1.39 (1.18-1.62) | 3.04E-03 |  |
|  | PCs (FinnGene) | MR Egger |  | 1.11 (0.50-2.45) | 8.04E-01 |  |
|  |  | Weighted median |  | 1.25 (0.89-1.74) | 1.96E-01 |  |
|  |  | IVW |  | 1.37 (1.06-1.77) | 1.44E-02 |  |
|  |  | Simple mode |  | 1.23 (0.77-1.96) | 4.14E-01 |  |
|  |  | Weighted mode |  | 1.20 (0.81-1.77) | 3.98E-01 |  |

^†^β (95% CI) represents the associations of each SD increase/decrease in TL with PCs.

Abbreviations: MR=Mendelian Randomization; TL=Telomere Length; PCs=Prostate cancers; No=number; SNP=single nucleotide polymorphism; OR=odds ratio; CI=confidence interval; IVW=inverse variance weighted.

**Table S4. Univariable MR pleiotropy and heterogeneity test for the association between TL and PCs**

| **Horizontal pleiotropy** | | | | | |
| --- | --- | --- | --- | --- | --- |
| **Exposure** | **Outcome** | **Method** | **Egger_intercept** | **Intercept_se** | **P_intercept_** |
| TL(MRC IEU GWAS) | PCs(IEU) | MR Egger | -0.001 | 0.003 | 6.81E-01 |
|  | PCs(FinnGene) |  | 0.006 | 0.005 | 1.69E-01 |
| TL(GWAS Validation) | PCs(IEU) |  | 0.006 | 0.021 | 7.90E-01 |
|  | PCs(FinnGene) |  | 0.014 | 0.025 | 5.93E-01 |
| **Heterogeneity test** | | | | | |
| **Exposure** | **Outcome** | **Method** | **Q statistic** | **Q_df** | **P_heterogeneity_** |
| TL(MRC IEU GWAS) | PCs(IEU) | IVW | 582.04 | 150 | 3.54E-52 |
|  |  | MR Egger | 581.38 | 149 | 2.28E-52 |
|  | PCs(FinnGene) | IVW | 209.98 | 136 | 2.73E-05 |
|  |  | MR Egger | 212.96 | 135 | 3.81E-05 |
| TL(GWAS Validation) | PCs(IEU) | IVW | 36.71 | 9 | 2.97E-05 |
|  |  | MR Egger | 36.36 | 8 | 1.51E-05 |
|  | PCs(FinnGene) | IVW | 3.565 | 9 | 9.38E-01 |
|  |  | MR Egger | 3.255 | 8 | 9.17E-01 |

Abbreviations: MR=Mendelian Randomization; TL=Telomere Length; PCs=Prostate cancers; IVW=inverse variance weighted.

**Table S5. Inversive MR for the interactive relationships between PCs and TL**

| **Exposure** | **Outcome** | **Method** | **No. of SNP** | **^†^β (95% CI)** | **P value** | **FDR q-value** |
| --- | --- | --- | --- | --- | --- | --- |
| PCs(ieu-b-85) | TL(ieu-b-4879) | MR Egger | 128 | 0.978 (0.957-1.000) | 5.63E-02 | NA |
|  |  | Weighted median |  | 0.998 (0.991-1.006) | 6.68E-01 | NA |
|  |  | IVW |  | 0.998 (0.986-1.009) | 6.95E-01 | 1 |
|  |  | Simple mode |  | 0.997 (0.984-1.011) | 6.70E-01 | NA |
|  |  | Weighted mode |  | 1.000 (0.991-1.009) | 9.17E-01 | NA |
| PCs(finn-b-C3_PROSTATE) | TL(ieu-b-4879) | MR Egger | 19 | 1.012 (0.973-1.054) | 5.50E-01 | NA |
|  |  | Weighted median |  | 1.000 (0.991-1.009) | 9.54E-01 | NA |
|  |  | IVW |  | 0.993 (0.975-1.011) | 4.50E-01 | 1 |
|  |  | Simple mode |  | 1.000 (0.988-1.012) | 9.84E-01 | NA |
|  |  | Weighted mode |  | 1.000 (0.990-1.010) | 9.79E-01 | NA |

^†^β (95% CI) represents the associations of each SD increase/decrease in PCs with TL.

Abbreviations: PCs=prostate cancers; TL=Telomere Length; CI=confidence interval; FDR=false discovery rate; IVW=inverse variance weighted; MR=Mendelian Randomization; NA=not applicable; No=number; SNP=single nucleotide polymorphism.

**Table S6. Univariable MR estimates for the causal effect of TL on potential mediators**

| **Outcome** | **Method** | **No. of SNP** | **^†^β (95% CI)**^†^ | **P value** | **FDR q-value** | **F-statistic** |
| --- | --- | --- | --- | --- | --- | --- |
| Alzheimer's_disease | MR Egger | 136 | 0.843 (0.590-1.206) | 3.52E-01 | NA | 119.818 |
|  | Weighted median |  | 0.809 (0.605-1.082) | 1.53E-01 | NA |  |
|  | IVW |  | 0.782 (0.641-0.955) | 1.60E-02 | 2.52E-02 |  |
|  | Simple mode |  | 0.796 (0.407-1.559) | 5.08E-01 | NA |  |
|  | Weighted mode |  | 0.663 (0.445-0.987) | 4.49E-02 | NA |  |
| Liver_iron_content | MR Egger | 144 | 0.935 (0.824-1.061) | 3.00E-01 | NA | 118.512 |
|  | Weighted median |  | 0.917 (0.835-1.006) | 6.79E-02 | NA |  |
|  | IVW |  | 0.933 (0.870-1.001) | 4.44E-02 | 4.25E-02 |  |
|  | Simple mode |  | 0.980 (0.800-1.200) | 8.44E-01 | NA |  |
|  | Weighted mode |  | 0.910 (0.820-1.009) | 7.59E-02 | NA |  |
| Hypertension | MR Egger | 136 | 1.023 (1.005-1.041) | 1.39E-02 | NA | 118.830 |
|  | Weighted median |  | 1.020 (1.006-1.034) | 6.02E-03 | NA |  |
|  | IVW |  | 1.021 (1.011-1.031) | 5.87E-05 | 2.97E-04 |  |
|  | Simple mode |  | 1.000 (0.972-1.028) | 9.97E-01 | NA |  |
|  | Weighted mode |  | 1.019 (1.003-1.034) | 2.10E-02 | NA |  |
| Coronary_heart_disease | MR Egger | 52 | 0.578 (0.368-0.908) | 2.13E-02 | NA | 119.438 |
|  | Weighted median |  | 0.722 (0.535-0.974) | 3.31E-02 | NA |  |
|  | IVW |  | 0.739 (0.596-0.917) | 6.09E-03 | 1.23E-02 |  |
|  | Simple mode |  | 0.894 (0.520-1.537) | 6.88E-01 | NA |  |
|  | Weighted mode |  | 0.619 (0.427-0.898) | 1.45E-02 | NA |  |
| Mean_corpuscular_hemoglobin | MR Egger | 145 | 0.782 (0.643-0.952) | 1.55E-02 | NA | 117.844 |
|  | Weighted median |  | 0.855 (0.811-0.901) | 6.35E-09 | NA |  |
|  | IVW |  | 0.820 (0.735-0.915) | 4.06E-04 | 1.37E-03 |  |
|  | Simple mode |  | 0.885 (0.787-0.995) | 4.30E-02 | NA |  |
|  | Weighted mode |  | 0.846 (0.794-0.903) | 1.22E-06 | NA |  |
| Arm fat percentage (right) | MR Egger | 139 | 0.983 (0.935-1.033) | 4.94E-01 | NA | 117.871 |
|  | Weighted median |  | 0.984 (0.963-1.006) | 1.48E-01 | NA |  |
|  | IVW |  | 0.972 (0.945-1.000) | 4.74E-02 | 4.36E-02 |  |
|  | Simple mode |  | 0.982 (0.937-1.028) | 4.30E-01 | NA |  |
|  | Weighted mode |  | 0.982 (0.960-1.004) | 1.10E-01 | NA |  |
| Body_fat_percentage | MR Egger | 136 | 0.968 (0.919-1.021) | 2.34E-01 | NA | 118.830 |
|  | Weighted median |  | 0.974 (0.949-1.000) | 4.61E-02 | NA |  |
|  | IVW |  | 0.966 (0.938-0.995) | 2.20E-02 | 3.05E-02 |  |
|  | Simple mode |  | 0.952 (0.899-1.009) | 1.01E-01 | NA |  |
|  | Weighted mode |  | 0.965 (0.939-0.992) | 1.30E-02 | NA |  |
| Sex_hormone_binding_globulin_levels | MR Egger | 145 | 0.937 (0.911-0.963) | 1.04E-05 | NA | 117.944 |
|  | Weighted median |  | 0.945 (0.933-0.957) | 8.03E-19 | NA |  |
|  | IVW |  | 0.962 (0.947-0.978) | 2.46E-06 | 2.49E-05 |  |
|  | Simple mode |  | 0.961 (0.936-0.987) | 3.70E-03 | NA |  |
|  | Weighted mode |  | 0.946 (0.935-0.957) | 6.77E-17 | NA |  |
| Naive_CD4-CD8-_T_cell %T_cell | MR Egger | 135 | 1.231 (0.929-1.631) | 1.50E-01 | NA | 119.281 |
|  | Weighted median |  | 1.257 (0.984-1.606) | 6.76E-02 | NA |  |
|  | IVW |  | 1.188 (1.014-1.391) | 3.25E-02 | 3.72E-02 |  |
|  | Simple mode |  | 1.135 (0.713-1.807) | 5.94E-01 | NA |  |
|  | Weighted mode |  | 1.212 (0.931-1.577) | 1.55E-01 | NA |  |
| Arm fat percentage (left) | MR Egger | 136 | 0.982 (0.933-1.034) | 5.01E-01 | NA | 118.830 |
|  | Weighted median |  | 0.987 (0.963-1.012) | 2.91E-01 | NA |  |
|  | IVW |  | 0.970 (0.942-0.998) | 3.88E-02 | 4.03E-02 |  |
|  | Simple mode |  | 0.982 (0.932-1.035) | 4.93E-01 | NA |  |
|  | Weighted mode |  | 0.977 (0.951-1.004) | 1.00E-01 | NA |  |
| Circulating_leptin_levels | MR Egger | 27 | 0.827 (0.683-1.002) | 6.44E-02 | NA | 196.902 |
|  | Weighted median |  | 0.856 (0.753-0.973) | 1.76E-02 | NA |  |
|  | IVW |  | 0.857 (0.768-0.957) | 5.93E-03 | 1.21E-02 |  |
|  | Simple mode |  | 0.936 (0.724-1.211) | 6.20E-01 | NA |  |
|  | Weighted mode |  | 0.873 (0.768-0.992) | 4.71E-02 | NA |  |
| Epstein_Barr_virus_antibody_levels | MR Egger | 141 | 1.073 (0.857-1.343) | 5.42E-01 | NA | 119.705 |
|  | Weighted median |  | 1.138 (0.940-1.378) | 1.86E-01 | NA |  |
|  | IVW |  | 1.090 (0.962-1.235) | 1.77E-01 | 1.35E-01 |  |
|  | Simple mode |  | 1.196 (0.808-1.772) | 3.72E-01 | NA |  |
|  | Weighted mode |  | 1.087 (0.883-1.338) | 4.33E-01 | NA |  |
| Alcoholic_drinks_per_week | MR Egger | 136 | 0.994 (0.958-1.032) | 7.56E-01 | NA | 117.798 |
|  | Weighted median |  | 1.001 (0.975-1.027) | 9.65E-01 | NA |  |
|  | IVW |  | 0.994 (0.973-1.015) | 5.60E-01 | 3.06E-01 |  |
|  | Simple mode |  | 1.004 (0.951-1.061) | 8.75E-01 | NA |  |
|  | Weighted mode |  | 0.990 (0.963-1.018) | 4.85E-01 | NA |  |
| Cigarettes_smoked_per_day | MR Egger | 136 | 1.030 (0.948-1.120) | 4.81E-01 | NA | 117.798 |
|  | Weighted median |  | 0.984 (0.913-1.062) | 6.84E-01 | NA |  |
|  | IVW |  | 0.977 (0.932-1.025) | 3.49E-01 | 2.19E-01 |  |
|  | Simple mode |  | 0.944 (0.798-1.117) | 5.03E-01 | NA |  |
|  | Weighted mode |  | 1.013 (0.934-1.100) | 7.54E-01 | NA |  |
| Fresh_tomato_intake | MR Egger | 139 | 0.985 (0.878-1.105) | 7.97E-01 | NA | 117.871 |
|  | Weighted median |  | 0.941 (0.849-1.043) | 2.45E-01 | NA |  |
|  | IVW |  | 0.989 (0.927-1.055) | 7.37E-01 | 3.67E-01 |  |
|  | Simple mode |  | 0.893 (0.707-1.128) | 3.44E-01 | NA |  |
|  | Weighted mode |  | 0.947 (0.847-1.059) | 3.40E-01 | NA |  |
| Lifetime_number_of_sexual_partners | MR Egger | 138 | 1.013 (0.974-1.054) | 5.13E-01 | NA | 117.871 |
|  | Weighted median |  | 1.004 (0.980-1.028) | 7.54E-01 | NA |  |
|  | IVW |  | 1.003 (0.981-1.025) | 8.11E-01 | 3.89E-01 |  |
|  | Simple mode |  | 0.993 (0.938-1.051) | 8.06E-01 | NA |  |
|  | Weighted mode |  | 1.004 (0.977-1.031) | 7.98E-01 | NA |  |
| Vitamin_E | MR Egger | 139 | 1.041 (0.962-1.126) | 3.24E-01 | NA | 117.871 |
|  | Weighted median |  | 1.023 (0.955-1.096) | 5.15E-01 | NA |  |
|  | IVW |  | 1.025 (0.981-1.071) | 2.75E-01 | 1.85E-01 |  |
|  | Simple mode |  | 1.071 (0.921-1.245) | 3.74E-01 | NA |  |
|  | Weighted mode |  | 0.992 (0.924-1.066) | 8.36E-01 | NA |  |
| Vitamin_D | MR Egger | 122 | 0.999 (0.993-1.005) | 6.51E-01 | NA | 121.865 |
|  | Weighted median |  | 0.996 (0.990-1.001) | 1.47E-01 | NA |  |
|  | IVW |  | 0.997 (0.994-1.001) | 1.36E-01 | 1.10E-01 |  |
|  | Simple mode |  | 0.995 (0.983-1.006) | 3.55E-01 | NA |  |
|  | Weighted mode |  | 0.996 (0.989-1.004) | 3.42E-01 | NA |  |
| Saturated_fatty_acids | MR Egger | 147 | 0.997 (0.923-1.078) | 9.49E-01 | NA | 116.693 |
|  | Weighted median |  | 1.013 (0.964-1.064) | 6.13E-01 | NA |  |
|  | IVW |  | 1.019 (0.976-1.064) | 3.96E-01 | 2.37E-01 |  |
|  | Simple mode |  | 1.019 (0.913-1.137) | 7.43E-01 | NA |  |
|  | Weighted mode |  | 1.028 (0.973-1.086) | 3.30E-01 | NA |  |
| Polyunsaturated_fatty_acids | MR Egger | 147 | 0.984 (0.888-1.091) | 7.59E-01 | NA | 116.693 |
|  | Weighted median |  | 1.000 (0.950-1.054) | 9.86E-01 | NA |  |
|  | IVW |  | 1.020 (0.963-1.081) | 4.98E-01 | 2.81E-01 |  |
|  | Simple mode |  | 0.936 (0.849-1.033) | 1.92E-01 | NA |  |
|  | Weighted mode |  | 0.988 (0.939-1.040) | 6.49E-01 | NA |  |
| Coffee_intake | MR Egger | 139 | 1.013 (0.985-1.043) | 3.67E-01 | NA | 117.871 |
|  | Weighted median |  | 0.997 (0.975-1.019) | 7.85E-01 | NA |  |
|  | IVW |  | 1.002 (0.986-1.018) | 8.13E-01 | 3.90E-01 |  |
|  | Simple mode |  | 0.979 (0.917-1.045) | 5.24E-01 | NA |  |
|  | Weighted mode |  | 1.001 (0.975-1.028) | 9.54E-01 | NA |  |
| Fried_potatoes_intake | MR Egger | 138 | 0.981 (0.900-1.069) | 6.63E-01 | NA | 117.871 |
|  | Weighted median |  | 0.999 (0.927-1.077) | 9.81E-01 | NA |  |
|  | IVW |  | 0.991 (0.945-1.040) | 7.28E-01 | 3.64E-01 |  |
|  | Simple mode |  | 1.024 (0.878-1.194) | 7.64E-01 | NA |  |
|  | Weighted mode |  | 0.993 (0.918-1.075) | 8.62E-01 | NA |  |
| Total_fatty_acids | MR Egger | 147 | 1.000 (0.916-1.093) | 9.95E-01 | NA | 116.693 |
|  | Weighted median |  | 1.020 (0.968-1.076) | 4.58E-01 | NA |  |
|  | IVW |  | 1.028 (0.978-1.080) | 2.75E-01 | 1.85E-01 |  |
|  | Simple mode |  | 1.012 (0.897-1.143) | 8.46E-01 | NA |  |
|  | Weighted mode |  | 1.024 (0.974-1.076) | 3.56E-01 | NA |  |

^†^β (95% CI) represents the associations of each SD increase/decrease in TL with each potential mediator.

Abbreviations: PCs=prostate cancers; IVW=inverse variance weighted; MR=Mendelian Randomization; NA=not applicable; OR=odds ratio; FDR=false discovery rate; SNP=single nucleotide polymorphism.

**Table S7. Univariable MR pleiotropy and heterogeneity test for the associations between TL and potential mediators**

| **Horizontal pleiotropy test** | | | | |
| --- | --- | --- | --- | --- |
| **Exposure** | **Method** | **Egger_intercept** | **Intercept_se** | **P_intercept_** |
| Alzheimer's_disease | MR Egger | -2.60E-03 | 5.20E-03 | 6.19E-01 |
| Liver_iron_content | MR Egger | -7.82E-05 | 1.80E-03 | 9.65E-01 |
| Hypertension | MR Egger | -7.00E-05 | 3.00E-04 | 7.82E-01 |
| Coronary_heart_disease | MR Egger | 6.70E-03 | 5.50E-03 | 2.31E-01 |
| Mean_corpuscular_hemoglobin | MR Egger | 1.60E-03 | 2.80E-03 | 5.70E-01 |
| Arm fat percentage (right) | MR Egger | -3.69E-04 | 7.14E-04 | 6.06E-01 |
| Body_fat_percentage | MR Egger | -8.74E-05 | 8.00E-04 | 9.08E-01 |
| Sex_hormone_binding_globulin_levels | MR Egger | 9.00E-04 | 4.00E-04 | 2.40E-01 |
| Naive_CD4-CD8-_T_cell %T_cell | MR Egger | -1.20E-03 | 4.20E-03 | 7.65E-01 |
| Arm fat percentage (left) | MR Egger | -4.38E-04 | 7.37E-04 | 5.53E-01 |
| Circulating_leptin_levels | MR Egger | 1.56E-03 | 3.51E-03 | 6.61E-01 |
| Epstein_Barr_virus_antibody_levels | MR Egger | 5.00E-04 | 3.20E-03 | 8.67E-01 |
| Alcoholic_drinks_per_week | MR Egger | -1.28E-05 | 5.20E-04 | 9.81E-01 |
| Cigarettes_smoked_per_day | MR Egger | -1.81E-03 | 1.20E-03 | 1.31E-01 |
| Fresh_tomato_intake | MR Egger | 1.40E-04 | 1.60E-03 | 9.34E-01 |
| Lifetime_number_of_sexual_partners | MR Egger | -3.50E-04 | 5.60E-04 | 5.29E-01 |
| Vitamin_E | MR Egger | -5.10E-04 | 1.10E-03 | 6.49E-01 |
| Vitamin_D | MR Egger | -3.81E-05 | 8.28E-05 | 6.64E-01 |
| Saturated_fatty_acids | MR Egger | 7.20E-04 | 1.10E-03 | 5.15E-01 |
| Polyunsaturated_fatty_acids | MR Egger | 1.22E-03 | 1.50E-03 | 4.06E-01 |
| Coffee_intake | MR Egger | -3.80E-04 | 4.10E-04 | 3.15E-01 |
| Fried_potatoes_intake | MR Egger | 3.60E-04 | 1.20E-03 | 7.71E-01 |
| Total_fatty_acids | MR Egger | 9.20E-04 | 1.30E-03 | 4.64E-01 |
| **Heterogeneity test** | | | | |
| **Exposure** | **Method** | **Q statistic** | **Q_df** | **P_heterogeneity_** |
| Alzheimer's_disease | IVW | 178.951 | 135 | 6.77E-03 |
|  | MR Egger | 178.620 | 134 | 6.03E-03 |
| Liver_iron_content | IVW | 217.373 | 143 | 6.04E-05 |
|  | MR Egger | 217.370 | 142 | 4.82E-05 |
| Hypertension | IVW | 198.230 | 135 | 3.24E-04 |
|  | MR Egger | 198.116 | 134 | 2.67E-04 |
| Coronary_heart_disease | IVW | 65.887 | 51 | 8.81E-02 |
|  | MR Egger | 64.002 | 50 | 7.84E-02 |
| Mean_corpuscular_hemoglobin | IVW | 2503.939 | 144 | 0.00E+00 |
|  | MR Egger | 2498.267 | 143 | 0.00E+00 |
| Arm fat percentage (right) | IVW | 805.571 | 138 | 8.00E-95 |
|  | MR Egger | 804.002 | 137 | 6.32E-95 |
| Arm fat percentage (left) | IVW | 581.894 | 135 | 8.93E-57 |
|  | MR Egger | 580.364 | 134 | 7.72E-57 |
| Body_fat_percentage | IVW | 587.849 | 135 | 8.93E-58 |
|  | MR Egger | 587.790 | 134 | 4.34E-58 |
| Sex_hormone_binding_globulin_levels | IVW | 768.842 | 144 | 5.35E-86 |
|  | MR Egger | 741.856 | 143 | 1.35E-81 |
| Naive_CD4-CD8-_T_cell %T_cell | IVW | 131.945 | 134 | 5.34E-01 |
|  | MR Egger | 131.855 | 133 | 5.12E-01 |
| Circulating_leptin_levels | IVW | 40.098 | 26 | 3.82E-02 |
|  | MR Egger | 39.784 | 25 | 3.07E-02 |
| Epstein_Barr_virus_antibody_levels | IVW | 157.552 | 140 | 1.47E-01 |
|  | MR Egger | 157.520 | 139 | 1.35E-01 |
| Alcoholic_drinks_per_week | IVW | 271.842 | 135 | 2.96E-11 |
|  | MR Egger | 271.841 | 134 | 2.06E-11 |
| Cigarettes_smoked_per_day | IVW | 171.894 | 135 | 1.75E-02 |
|  | MR Egger | 168.988 | 134 | 2.19E-02 |
| Fresh_tomato_intake | IVW | 154.703 | 138 | 1.57E-01 |
|  | MR Egger | 154.695 | 137 | 1.43E-01 |
| Lifetime_number_of_sexual_partners | IVW | 341.367 | 137 | 1.93E-19 |
|  | MR Egger | 340.371 | 136 | 1.64E-19 |
| Vitamin_E | IVW | 155.249 | 138 | 1.50E-01 |
|  | MR Egger | 155.014 | 137 | 1.39E-01 |
| Vitamin_D | IVW | 130.588 | 121 | 2.60E-01 |
|  | MR Egger | 130.358 | 120 | 2.44E-01 |
| Saturated_fatty_acids | IVW | 299.141 | 146 | 1.33E-12 |
|  | MR Egger | 298.263 | 145 | 1.16E-12 |
| Polyunsaturated_fatty_acids | IVW | 545.125 | 146 | 2.12E-47 |
|  | MR Egger | 542.530 | 145 | 2.84E-47 |
| Coffee_intake | IVW | 241.324 | 138 | 1.26E-07 |
|  | MR Egger | 239.794 | 137 | 1.32E-07 |
| Fried_potatoes_intake | IVW | 143.441 | 137 | 3.36E-01 |
|  | MR Egger | 143.351 | 136 | 3.16E-01 |
| Total_fatty_acids | IVW | 388.245 | 146 | 7.04E-24 |
|  | MR Egger | 386.807 | 145 | 6.74E-24 |

Abbreviations: PCs=prostate cancers; IVW=inverse variance weighted; MR=Mendelian Randomization; NA=not applicable; SE=standard error.

**Table S8. Inversive MR for the interactive relationships between TL and potential mediators**

| **Exposure** | **Method** | **No. of SNP** | **^†^β (95% CI)**^†^ | **P value** | **FDR q-value** | **F-statistic** |
| --- | --- | --- | --- | --- | --- | --- |
| Alzheimer's_disease | MR Egger | 6 | 1.017 (1.007-1.028) | 3.36E-02 | NA | 265.120 |
|  | Weighted median |  | 1.012 (1.008-1.017) | 7.38E-07 | NA |  |
|  | IVW |  | 1.010 (1.001-1.018) | 2.21E-02 | 2.56E-02 |  |
|  | Simple mode |  | 1.010 (0.997-1.024) | 2.03E-01 | NA |  |
|  | Weighted mode |  | 1.013 (1.008-1.017) | 3.28E-03 | NA |  |
| Liver_iron_content | MR Egger | 9 | 1.008 (0.973-1.045) | 6.68E-01 | NA | 252.353 |
|  | Weighted median |  | 1.015( 1.002-1.028) | 2.37E-02 | NA |  |
|  | IVW |  | 1.018 (0.993-1.043) | 1.60E-01 | 1.19E-01 |  |
|  | Simple mode |  | 0.998 (0.973-1.025) | 9.02E-01 | NA |  |
|  | Weighted mode |  | 1.014 (1.001-1.026) | 6.71E-02 | NA |  |
| Hypertension | MR Egger | 6 | 1.347 (0.124-14.658) | 8.19E-01 | NA | 40.060 |
|  | Weighted median |  | 1.153 (0.864-1.539) | 3.34E-01 | NA |  |
|  | IVW |  | 1.037 (0.820-1.311) | 7.61E-01 | 3.91E-01 |  |
|  | Simple mode |  | 1.171 (0.749-1.830) | 5.19E-01 | NA |  |
|  | Weighted mode |  | 1.171 (0.758-1.810) | 5.08E-01 | NA |  |
| Coronary_heart_disease | MR Egger | 15 | 1.014 (0.954-1.078) | 6.68E-01 | NA | 45.353 |
|  | Weighted median |  | 1.000 (0.988-1.013) | 9.73E-01 | NA |  |
|  | IVW |  | 0.995 (0.974-1.015) | 6.00E-01 | 3.36E-01 |  |
|  | Simple mode |  | 0.998 (0.981-1.016) | 8.51E-01 | NA |  |
|  | Weighted mode |  | 0.998 (0.985-1.012) | 8.00E-01 | NA |  |
| Mean_corpuscular_hemoglobin | MR Egger | 201 | 0.983 (0.945-1.023) | 4.00E-01 | NA | 147.038 |
|  | Weighted median |  | 0.992 (0.978-1.005) | 2.11E-01 | NA |  |
|  | IVW |  | 0.972 (0.950-0.995) | 1.53E-02 | 2.19E-02 |  |
|  | Simple mode |  | 0.987 (0.962-1.012) | 3.09E-01 | NA |  |
|  | Weighted mode |  | 0.999 (0.987-1.010) | 8.24E-01 | NA |  |
| Arm fat percentage (right) | MR Egger | 372 | 0.906 (0.832-0.987) | 2.47E-02 | NA | 58.611 |
|  | Weighted median |  | 0.935 (0.907-0.964) | 1.50E-05 | NA |  |
|  | IVW |  | 0.928 (0.902-0.955) | 3.76E-07 | 3.44E-06 |  |
|  | Simple mode |  | 0.942 (0.866-1.023) | 1.57E-01 | NA |  |
|  | Weighted mode |  | 0.934 (0.884-0.988) | 1.72E-02 | NA |  |
| Body_fat_percentage | MR Egger | 249 | 0.912 (0.813-1.023) | 1.17E-01 | NA | 52.366 |
|  | Weighted median |  | 0.953 (0.925-0.982) | 1.91E-03 | NA |  |
|  | IVW |  | 0.925 (0.896-0.956) | 2.31E-06 | 1.06E-05 |  |
|  | Simple mode |  | 0.956 (0.874-1.046) | 3.27E-01 | NA |  |
|  | Weighted mode |  | 0.956 (0.892-1.024) | 2.02E-01 | NA |  |
| Sex_hormone_binding_globulin_levels | MR Egger | 369 | 1.032 (0.933-1.141) | 5.46E-01 | NA | 118.982 |
|  | Weighted median |  | 0.982 (0.947-1.019) | 3.45E-01 | NA |  |
|  | IVW |  | 0.978 (0.925-1.035) | 4.49E-01 | 2.75E-01 |  |
|  | Simple mode |  | 0.980 (0.895-1.073) | 6.59E-01 | NA |  |
|  | Weighted mode |  | 0.980 (0.934-1.027) | 3.98E-01 | NA |  |
| Naive_CD4-CD8-_T_cell %T_cell | Wald Ratio | 1 | 0.978 (0.950-1.007) | 1.39E-01 | 1.06E-01 | 33.960 |
| Arm fat percentage (left) | MR Egger | 248 | 0.947 (0.859-1.044) | 2.77E-01 | NA | 53.360 |
|  | Weighted median |  | 0.954 (0.925-0.983) | 2.13E-03 | NA |  |
|  | IVW |  | 0.933 (0.905-0.962) | 7.12E-06 | 2.17E-05 |  |
|  | Simple mode |  | 0.975 (0.897-1.060) | 5.59E-01 | NA |  |
|  | Weighted mode |  | 0.949 (0.890-1.013) | 1.18E-01 | NA |  |
| Circulating_leptin_levels | MR Egger | 3 | 0.691 (0.449-1.065) | 3.43E-01 | NA | 56.186 |
|  | Weighted median |  | 0.968 (0.913-1.027) | 2.78E-01 | NA |  |
|  | IVW |  | 0.971 (0.905-1.043) | 4.22E-01 | 2.62E-01 |  |
|  | Simple mode |  | 0.973 (0.892-1.062) | 6.07E-01 | NA |  |
|  | Weighted mode |  | 0.960 (0.887-1.038) | 4.14E-01 | NA |  |
| Epstein_Barr_virus_antibody_levels | IVW | 2 | 1.016 (1.000-1.031) | 4.75E-02 | 4.57E-02 | 103.181 |
| Alcoholic_drinks_per_week | MR Egger | 35 | 0.930 (0.844-1.026) | 1.56E-01 | NA | 76.220 |
|  | Weighted median |  | 0.921 (0.869-0.977) | 6.56E-03 | NA |  |
|  | IVW |  | 0.933 (0.879-0.990) | 2.26E-02 | 2.58E-02 |  |
|  | Simple mode |  | 0.955 (0.842-1.083) | 4.81E-01 | NA |  |
|  | Weighted mode |  | 0.926 (0.870-0.985) | 2.06E-02 | NA |  |
| Cigarettes_smoked_per_day | MR Egger | 22 | 0.983 (0.940-1.027) | 4.42E-01 | NA | 100.147 |
|  | Weighted median |  | 0.972 (0.950-0.994) | 1.13E-02 | NA |  |
|  | IVW |  | 0.976 (0.953-1.001) | 5.54E-02 | 5.07E-02 |  |
|  | Simple mode |  | 0.974 (0.906-1.046) | 4.76E-01 | NA |  |
|  | Weighted mode |  | 0.977 (0.954-1.000) | 6.47E-02 | NA |  |
| Fresh_tomato_intake | NA | NA | NA | NA | NA | NA |
| Lifetime_number_of_sexual_partners | MR Egger | 60 | 1.195 (0.837-1.704) | 3.31E-01 | NA | 38.415 |
|  | Weighted median |  | 0.992 (0.928-1.060) | 8.11E-01 | NA |  |
|  | IVW |  | 1.007 (0.932-1.087) | 8.61E-01 | 4.21E-01 |  |
|  | Simple mode |  | 1.019 (0.851-1.219) | 8.42E-01 | NA |  |
|  | Weighted mode |  | 1.040 (0.878-1.233) | 6.49E-01 | NA |  |
| Vitamin_E | NA | NA | NA | NA | NA | NA |
| Vitamin_D | NA | NA | NA | NA | NA | NA |
| Saturated_fatty_acids | MR Egger | 51 | 1.039 (1.005-1.075) | 3.03E-02 | NA | 118.266 |
|  | Weighted median |  | 1.014 (0.992-1.037) | 2.13E-01 | NA |  |
|  | IVW |  | 1.017 (0.998-1.037) | 8.62E-02 | 7.18E-02 |  |
|  | Simple mode |  | 1.022 (0.982-1.063) | 2.92E-01 | NA |  |
|  | Weighted mode |  | 1.014 (0.982-1.047) | 4.01E-01 | NA |  |
| Polyunsaturated_fatty_acids | MR Egger | 60 | 1.036 (1.004-1.069) | 3.05E-02 | NA | 134.815 |
|  | Weighted median |  | 1.007 (0.989-1.025) | 4.29E-01 | NA |  |
|  | IVW |  | 1.023 (1.006-1.040) | 8.01E-03 | 1.54E-02 |  |
|  | Simple mode |  | 1.015 (0.983-1.048) | 3.62E-01 | NA |  |
|  | Weighted mode |  | 1.005 (0.981-1.029) | 7.07E-01 | NA |  |
| Coffee_intake | MR Egger | 38 | 0.878 (0.758-1.018) | 9.32E-02 | NA | 72.712 |
|  | Weighted median |  | 0.925 (0.857-0.999) | 4.85E-02 | NA |  |
|  | IVW |  | 0.963 (0.894-1.038) | 3.27E-01 | 2.16E-01 |  |
|  | Simple mode |  | 0.927 (0.779-1.103) | 3.96E-01 | NA |  |
|  | Weighted mode |  | 0.923 (0.850-1.002) | 6.31E-02 | NA |  |
| Fried_potatoes_intake | Wald Ratio | 1 | 1.008 (0.903-1.124) | 8.89E-01 | 4.28E-01 | 33.362 |
| Total_fatty_acids | MR Egger | 58 | 1.029 (1.001-1.059) | 5.05E-02 | NA | 123.891 |
|  | Weighted median |  | 1.019 (1.000-1.039) | 5.57E-02 | NA |  |
|  | IVW |  | 1.020 (1.003-1.037) | 1.98E-02 | 2.45E-02 |  |
|  | Simple mode |  | 1.026 (0.991-1.063) | 1.52E-01 | NA |  |
|  | Weighted mode |  | 1.012 (0.983-1.041) | 4.19E-01 | NA |  |

^†^β (95% CI) represents the associations of each SD increase/decrease in each potential mediator with TL.

Abbreviations: PCs=prostate cancers; TL=Telomere Length; CI=confidence interval; FDR=false discovery rate; IVW=inverse variance weighted; MR=Mendelian Randomization; NA=not applicable; No=number; SNP=single nucleotide polymorphism.

**Table S9. Inversive MR pleiotropy and heterogeneity test for the associations between TL and potential mediators**

| **Horizontal pleiotropy test** | | | | |
| --- | --- | --- | --- | --- |
| **Exposure** | **Method** | **Egger_intercept** | **Intercept_se** | **P_intercept_** |
| Alzheimer's_disease | MR Egger | -5.57E-03 | 3.08E-03 | 1.45E-01 |
| Liver_iron_content | MR Egger | 2.23E-03 | 3.09E-03 | 4.92E-01 |
| Hypertension | MR Egger | -1.92E-03 | 8.89E-03 | 8.40E-01 |
| Coronary_heart_disease | MR Egger | -2.79E-03 | 4.26E-03 | 5.24E-01 |
| Mean_corpuscular_hemoglobin | MR Egger | -7.35E-04 | 1.07E-03 | 4.91E-01 |
| Arm fat percentage (right) | MR Egger | 3.38E-04 | 5.82E-04 | 5.62E-01 |
| Body_fat_percentage | MR Egger | 2.35E-04 | 9.00E-04 | 7.94E-01 |
| Sex_hormone_binding_globulin_levels | MR Egger | -7.97E-04 | 6.42E-04 | 2.15E-01 |
| Naive_CD4-CD8-_T_cell %T_cell | MR Egger | -1.24E-03 | 4.15E-03 | 7.65E-01 |
| Arm fat percentage (left) | MR Egger | -2.40E-04 | 7.55E-04 | 7.50E-01 |
| Circulating_leptin_levels | MR Egger | 1.74E-02 | 1.12E-02 | 3.64E-01 |
| Epstein_Barr_virus_antibody_levels | MR Egger | NA | NA | NA |
| Alcoholic_drinks_per_week | MR Egger | 7.77E-05 | 1.00E-03 | 9.39E-01 |
| Cigarettes_smoked_per_day | MR Egger | -5.05E-04 | 1.47E-03 | 7.34E-01 |
| Fresh_tomato_intake | MR Egger | NA | NA | NA |
| Lifetime_number_of_sexual_partners | MR Egger | -2.29E-03 | 2.37E-03 | 3.38E-01 |
| Vitamin_E | MR Egger | NA | NA | NA |
| Vitamin_D | MR Egger | NA | NA | NA |
| Saturated_fatty_acids | MR Egger | -1.37E-03 | 9.09E-04 | 1.37E-01 |
| Polyunsaturated_fatty_acids | MR Egger | -9.05E-04 | 9.55E-04 | 3.47E-01 |
| Coffee_intake | MR Egger | 1.75E-03 | 1.23E-03 | 1.65E-01 |
| Fried_potatoes_intake | MR Egger | NA | NA | NA |
| Total_fatty_acids | MR Egger | -6.38E-04 | 8.00E-04 | 4.28E-01 |
| **Heterogeneity test** | | | | |
| **Outcome** | **Method** | **Q statistic** | **Q_df** | **P_heterogeneity_** |
| Alzheimer's_disease | IVW | 16.269 | 5 | 6.12E-03 |
|  | MR Egger | 8.957 | 4 | 6.22E-02 |
| Liver_iron_content | IVW | 38.319 | 8 | 6.57E-06 |
|  | MR Egger | 35.648 | 7 | 8.45E-06 |
| Hypertension | IVW | 4.139 | 5 | 5.30E-01 |
|  | MR Egger | 4.092 | 4 | 3.94E-01 |
| Coronary_heart_disease | IVW | 73.928 | 14 | 3.72E-10 |
|  | MR Egger | 71.571 | 13 | 4.12E-10 |
| Mean_corpuscular_hemoglobin | IVW | 2539.095 | 200 | 0.00E+00 |
|  | MR Egger | 2533.039 | 199 | 0.00E+00 |
| Arm fat percentage (right) | IVW | 1001.169 | 371 | 2.34E-59 |
|  | MR Egger | 1000.259 | 370 | 1.90E-59 |
| Body_fat_percentage | IVW | 754.175 | 248 | 1.67E-52 |
|  | MR Egger | 753.967 | 247 | 1.02E-52 |
| Sex_hormone_binding_globulin_levels | IVW | 2916.306 | 368 | 0.00E+00 |
|  | MR Egger | 2904.089 | 367 | 0.00E+00 |
| Naive_CD4-CD8-_T_cell %T_cell | IVW | 131.945 | 134 | 5.34E-01 |
|  | MR Egger | 131.855 | 133 | 5.12E-01 |
| Arm fat percentage (left) | IVW | 652.317 | 247 | 2.57E-38 |
|  | MR Egger | 652.048 | 246 | 1.71E-38 |
| Circulating_leptin_levels | IVW | 4.915 | 2 | 8.56E-02 |
|  | MR Egger | 1.441 | 1 | 2.30E-01 |
| Epstein_Barr_virus_antibody_levels | IVW | 0.433 | 1 | 5.11E-01 |
| Alcoholic_drinks_per_week | IVW | 80.523 | 34 | 1.21E-05 |
|  | MR Egger | 80.508 | 33 | 7.56E-06 |
| Cigarettes_smoked_per_day | IVW | 56.558 | 21 | 4.18E-05 |
|  | MR Egger | 56.225 | 20 | 2.69E-05 |
| Fresh_tomato_intake | NA | NA | NA | NA |
| Lifetime_number_of_sexual_partners | IVW | 175.336 | 59 | 1.78E-13 |
|  | MR Egger | 172.560 | 58 | 2.61E-13 |
| Vitamin_E | MR Egger | 652.048 | 246 | 1.71E-38 |
| Vitamin_D | NA | NA | NA | NA |
| Saturated_fatty_acids | IVW | 115.029 | 50 | 4.90E-07 |
|  | MR Egger | 109.898 | 49 | 1.44E-06 |
| Polyunsaturated_fatty_acids | IVW | 141.714 | 59 | 9.26E-09 |
|  | MR Egger | 139.554 | 58 | 1.13E-08 |
| Coffee_intake | IVW | 97.499 | 37 | 2.39E-07 |
|  | MR Egger | 92.344 | 36 | 7.63E-07 |
| Fried_potatoes_intake | NA | NA | NA | NA |
| Total_fatty_acids | IVW | 115.676 | 57 | 7.12E-06 |
|  | MR Egger | 114.375 | 56 | 6.92E-06 |

Abbreviations: TL=Telomere Length; IVW=inverse variance weighted; MR=Mendelian Randomization.

**Table S10. Univariable MR estimates for the causal effect of potential mediators on PCs**

| **Exposure** | **Method** | **No. of SNP** | **^†^β (95% CI)**^†^ | **P value** | **FDR q-value** | **F-statistic** |
| --- | --- | --- | --- | --- | --- | --- |
| Alzheimer's_disease | MR Egger | 6 | 0.961 (0.913-1.013) | 2.12E-01 | NA | 265.120 |
|  | Weighted median |  | 0.963 (0.944-0.983) | 3.27E-04 | NA |  |
|  | IVW |  | 0.961 (0.932-0.991) | 1.12E-02 | 2.48E-02 |  |
|  | Simple mode |  | 0.999 (0.948-1.052) | 9.71E-01 | NA |  |
|  | Weighted mode |  | 0.964 (0.944-0.985) | 2.02E-02 | NA |  |
| Liver_iron_content | MR Egger | 9 | 0.933 (0.869-1.002) | 9.80E-02 | NA | 252.353 |
|  | Weighted median |  | 0.940 (0.891-0.992) | 2.50E-02 | NA |  |
|  | IVW |  | 0.945 (0.901-0.992) | 2.18E-02 | 2.54E-02 |  |
|  | Simple mode |  | 0.930 (0.849-1.018) | 1.54E-01 | NA |  |
|  | Weighted mode |  | 0.941 (0.890-0.995) | 6.65E-02 | NA |  |
| Sex_hormone_binding_globulin_levels | MR Egger | 367 | 0.931 (0.719-1.205) | 5.86E-01 | NA | 119.425 |
|  | Weighted median |  | 0.997 (0.851-1.168) | 9.71E-01 | NA |  |
|  | IVW |  | 0.852 (0.737-0.986) | 3.10E-02 | 2.58E-02 |  |
|  | Simple mode |  | 0.791 (0.518-1.208) | 2.78E-01 | NA |  |
|  | Weighted mode |  | 1.185 (0.934-1.505) | 1.64E-01 | NA |  |
| Naive_CD4-CD8-_T_cell %T_cell | Wald ratio | 1 | 1.156 (1.030-1.296) | 1.34E-02 | 2.48E-02 | 33.960 |
| Circulating_leptin_levels | MR Egger | 3 | 0.628 (0.140-2.827) | 6.53E-01 | NA | 56.186 |
|  | Weighted median |  | 0.871 (0.702-1.082) | 2.12E-01 | NA |  |
|  | IVW |  | 0.818 (0.682-0.981) | 3.03E-02 | 2.58E-02 |  |
|  | Simple mode |  | 0.872 (0.668-1.137) | 4.18E-01 | NA |  |
|  | Weighted mode |  | 0.872 (0.680-1.118) | 3.93E-01 | NA |  |
| Hypertension | MR Egger | 6 | 0.000 (0.000-10.666) | 1.79E-01 | NA | 40.060 |
|  | Weighted median |  | 1.201 (0.307-4.693) | 7.92E-01 | NA |  |
|  | IVW |  | 0.851 (0.179-4.046) | 8.39E-01 | 4.17E-01 |  |
|  | Simple mode |  | 1.309 (0.204-8.409) | 7.88E-01 | NA |  |
|  | Weighted mode |  | 1.245 (0.220-7.055) | 8.14E-01 | NA |  |
| Coronary_heart_disease | MR Egger | 15 | 1.072 (0.900-1.276) | 4.52E-01 | NA | 45.353 |
|  | Weighted median |  | 0.990 (0.934-1.049) | 7.29E-01 | NA |  |
|  | IVW |  | 1.017 (0.959-1.078) | 5.73E-01 | 3.29E-01 |  |
|  | Simple mode |  | 0.932 (0.837-1.037) | 2.16E-01 | NA |  |
|  | Weighted mode |  | 0.970 (0.902-1.043) | 4.27E-01 | NA |  |
| Body_fat_percentage | MR Egger | 249 | 0.741 (0.496-1.106) | 1.43E-01 | NA | 52.420 |
|  | Weighted median |  | 0.879 (0.777-0.994) | 4.02E-02 | NA |  |
|  | IVW |  | 0.955 (0.854-1.068) | 4.18E-01 | 2.63E-01 |  |
|  | Simple mode |  | 0.729 (0.467-1.137) | 1.64E-01 | NA |  |
|  | Weighted mode |  | 0.714 (0.505-1.010) | 5.80E-02 | NA |  |

^†^β (95% CI) represents the associations of each SD increase/decrease in each potential mediator with PCs.

Abbreviations: TL=Telomere Length; CI=confidence interval; FDR=false discovery rate; IVW=inverse variance weighted; MR=Mendelian Randomization; NA=not applicable; No=number; SNP=single nucleotide polymorphism.

**Table S11. Univariable MR pleiotropy and heterogeneity test for the associations between potential mediators and PCs**

| **Horizontal pleiotropy test** | | | | |
| --- | --- | --- | --- | --- |
| **Exposure** | **Method** | **Egger_intercept** | **Intercept_se** | **P_intercept_** |
| Alzheimer's_disease | MR Egger | -2.95E-04 | 1.51E-02 | 9.85E-01 |
| Liver_iron_content | MR Egger | 3.01E-03 | 5.94E-03 | 6.28E-01 |
| Sex_hormone_binding_globulin_levels | MR Egger | 2.22E-03 | 2.87E-03 | 4.19E-01 |
| Naive_CD4-CD8-_T_cell %T_cell | MR Egger | NA | NA | NA |
| Circulating_leptin_levels | MR Egger | 1.36E-02 | 3.92E-02 | 7.88E-01 |
| Hypertension | MR Egger | 8.30E-02 | 5.15E-02 | 1.83E-01 |
| Coronary_heart_disease | MR Egger | -7.64E-03 | 1.22E-02 | 5.43E-01 |
| Body_fat_percentage | MR Egger | 4.06E-03 | 3.14E-03 | 1.97E-01 |
| **Heterogeneity test** | | | | |
| **Outcome** | **Method** | **Q statistic** | **Q_df** | **P_heterogeneity_** |
| Alzheimer's_disease | IVW | 12.697 | 5 | 2.64E-02 |
|  | MR Egger | 12.696 | 4 | 1.29E-02 |
| Liver_iron_content | IVW | 7.726 | 8 | 4.61E-01 |
|  | MR Egger | 7.452 | 7 | 3.83E-01 |
|  | MR Egger | 555.870 | 246 | 5.13E-26 |
| Sex_hormone_binding_globulin_levels | IVW | 1145.781 | 366 | 3.24E-81 |
|  | MR Egger | 1143.728 | 365 | 3.68E-81 |
| Naive_CD4-CD8-_T_cell %T_cell | NA | NA | NA | NA |
| Circulating_leptin_levels | IVW | 0.725 | 2 | 6.96E-01 |
|  | MR Egger | 0.605 | 1 | 4.37E-01 |
| Hypertension | IVW | 12.999 | 5 | 2.34E-02 |
|  | MR Egger | 7.886 | 4 | 9.59E-02 |
| Coronary_heart_disease | IVW | 36.426 | 14 | 9.01E-04 |
|  | MR Egger | 35.365 | 13 | 7.44E-04 |
| Body_fat_percentage | IVW | 548.580 | 248 | 8.89E-25 |
|  | MR Egger | 544.887 | 247 | 1.65E-24 |

Abbreviations: TL=Telomere Length; IVW=inverse variance weighted; MR=Mendelian Randomization..

**Table S12. Inversive MR for the interactive relationships between potential mediators and PCs**

| **Outcome** | **Method** | **No. of SNP** | **^†^β (95% CI)**^†^ | **P value** | **FDR q-value** | **F-statistic** |
| --- | --- | --- | --- | --- | --- | --- |
| Alzheimer's_disease | MR Egger | 117 | 1.036 (0.941-1.142) | 4.72E-01 | NA | 104.868 |
|  | Weighted median |  | 0.995 (0.916-1.082) | 9.11E-01 | NA |  |
|  | IVW |  | 0.995 (0.945-1.048) | 8.50E-01 | 9.52E-01 |  |
|  | Simple mode |  | 0.937 (0.765-1.147) | 5.29E-01 | NA |  |
|  | Weighted mode |  | 1.062 (0.938-1.202) | 3.46E-01 | NA |  |
| Liver_iron_content | MR Egger | 121 | 0.992 (0.956-1.030) | 6.78E-01 | NA | 102.398 |
|  | Weighted median |  | 0.994 (0.967-1.021) | 6.41E-01 | NA |  |
|  | IVW |  | 0.991 (0.972-1.010) | 3.42E-01 | 8.90E-01 |  |
|  | Simple mode |  | 0.984 (0.933-1.039) | 5.68E-01 | NA |  |
|  | Weighted mode |  | 1.002 (0.968-1.037) | 9.16E-01 | NA |  |
| Hypertension | MR Egger | 118 | 1.001 (0.995-1.006) | 8.43E-01 | NA | 105.094 |
|  | Weighted median |  | 1.001 (0.997-1.005) | 6.99E-01 | NA |  |
|  | IVW |  | 1.000 (0.997-1.002) | 7.67E-01 | 9.48E-01 |  |
|  | Simple mode |  | 0.999 (0.991-1.007) | 7.61E-01 | NA |  |
|  | Weighted mode |  | 1.000 (0.996-1.005) | 8.87E-01 | NA |  |
| Coronary_heart_disease | MR Egger | 86 | 0.992 (0.913-1.078) | 8.55E-01 | NA | 105.853 |
|  | Weighted median |  | 1.028 (0.964-1.097) | 3.99E-01 | NA |  |
|  | IVW |  | 0.996 (0.956-1.038) | 8.66E-01 | 9.53E-01 |  |
|  | Simple mode |  | 1.060 (0.929-1.209) | 3.87E-01 | NA |  |
|  | Weighted mode |  | 1.026 (0.929-1.133) | 6.19E-01 | NA |  |
| Body_fat_percentage | MR Egger | 118 | 1.003 (0.988-1.018) | 6.87E-01 | NA | 105.094 |
|  | Weighted median |  | 1.002 (0.993-1.010) | 7.11E-01 | NA |  |
|  | IVW |  | 1.001 (0.993-1.008) | 8.75E-01 | 9.54E-01 |  |
|  | Simple mode |  | 1.004 (0.983-1.025) | 7.20E-01 | NA |  |
|  | Weighted mode |  | 0.998 (0.986-1.010) | 7.19E-01 | NA |  |
| Sex_hormone_binding_globulin_levels | MR Egger | 122 | 0.997 (0.981-1.014) | 7.25E-01 | NA | 103.313 |
|  | Weighted median |  | 0.995 (0.991-0.999) | 2.17E-02 | NA |  |
|  | IVW |  | 0.995 (0.987-1.004) | 2.54E-01 | 8.57E-01 |  |
|  | Simple mode |  | 0.995 (0.988-1.002) | 1.48E-01 | NA |  |
|  | Weighted mode |  | 0.993 (0.989-0.998) | 6.87E-03 | NA |  |
| Circulating_leptin_levels | MR Egger | 21 | 0.989 (0.938-1.043) | 6.95E-01 | NA | 177.429 |
|  | Weighted median |  | 0.981 (0.942-1.021) | 3.40E-01 | NA |  |
|  | IVW |  | 0.970 (0.942-0.999) | 4.38E-02 | 5.08E-01 |  |
|  | Simple mode |  | 0.989 (0.928-1.054) | 7.30E-01 | NA |  |
|  | Weighted mode |  | 0.989 (0.935-1.046) | 6.96E-01 | NA |  |
| Naive_CD4-CD8-_T_cell %T_cell | MR Egger | 117 | 0.999 (0.903-1.106) | 9.88E-01 | NA | 100.589 |
|  | Weighted median |  | 0.974 (0.899-1.054) | 5.12E-01 | NA |  |
|  | IVW |  | 1.002 (0.952-1.054) | 9.37E-01 | 9.57E-01 |  |
|  | Simple mode |  | 0.995 (0.828-1.196) | 9.58E-01 | NA |  |
|  | Weighted mode |  | 0.940 (0.832-1.061) | 3.19E-01 | NA |  |

^†^β (95% CI) represents the associations of each SD increase/decrease in PCs with each potential mediator.

Abbreviations: PCs=prostate cancers; TL=Telomere Length; CI=confidence interval; FDR=false discovery rate; IVW=inverse variance weighted; MR=Mendelian Randomization; NA=not applicable; No=number; SNP=single nucleotide polymorphism.

**Table S13. Inversive MR pleiotropy and heterogeneity test for the associations between potential mediators and PCs**

| **Horizontal pleiotropy test** | | | | |
| --- | --- | --- | --- | --- |
| **Exposure** | **Method** | **Egger_intercept** | **Intercept_se** | **P_intercept_** |
| Alzheimer's_disease | MR Egger | -4.91E-03 | 5.03E-03 | 3.31E-01 |
| Liver_iron_content | MR Egger | -1.61E-04 | 1.86E-03 | 9.31E-01 |
| Hypertension | MR Egger | -1.11E-04 | 2.69E-04 | 6.80E-01 |
| Coronary_heart_disease | MR Egger | 4.61E-04 | 4.00E-03 | 9.08E-01 |
| Body_fat_percentage | MR Egger | -2.83E-04 | 7.50E-04 | 7.07E-01 |
| Sex_hormone_binding_globulin_levels | MR Egger | -2.30E-04 | 8.31E-04 | 7.83E-01 |
| Circulating_leptin_levels | MR Egger | -3.42E-03 | 3.96E-03 | 3.99E-01 |
| Naive_CD4-CD8-_T_cell %T_cell | MR Egger | 3.05E-04 | 4.81E-03 | 9.50E-01 |
| **Heterogeneity test** | | | | |
| **Outcome** | **Method** | **Q statistic** | **Q_df** | **P_heterogeneity_** |
| Alzheimer's_disease | IVW | 122.061 | 116 | 3.32E-01 |
|  | MR Egger | 121.058 | 115 | 3.31E-01 |
| Liver_iron_content | IVW | 150.517 | 120 | 3.10E-02 |
|  | MR Egger | 150.508 | 119 | 2.69E-02 |
| Hypertension | IVW | 154.249 | 117 | 1.20E-02 |
|  | MR Egger | 154.022 | 116 | 1.05E-02 |
| Coronary_heart_disease | IVW | 96.970 | 85 | 1.76E-01 |
|  | MR Egger | 96.955 | 84 | 1.58E-01 |
| Body_fat_percentage | IVW | 404.256 | 117 | 2.79E-33 |
|  | MR Egger | 403.761 | 116 | 1.78E-33 |
| Sex_hormone_binding_globulin_levels | IVW | 2092.493 | 121 | 0.00E+00 |
|  | MR Egger | 2091.160 | 120 | 0.00E+00 |
| Circulating_leptin_levels | IVW | 25.103 | 20 | 1.98E-01 |
|  | MR Egger | 24.156 | 19 | 1.90E-01 |
| Naive_CD4-CD8-_T_cell %T_cell | IVW | 116.438 | 116 | 4.71E-01 |
|  | MR Egger | 116.434 | 115 | 4.45E-01 |

Abbreviations: TL=Telomere Length; IVW=inverse variance weighted; MR=Mendelian Randomization.

**Table S14. Multivariable MR estimates for the independent effect of TL on PCs with adjustment for other related factors**

| **Variable** | **Method** | **^†^β (95% CI)**^†^ | **SE** | **P value** | **MVMR** **Instrument** **validity** **test** | **MVMR**  **Heterogeneity** **test** | | **MVMR** **directional** **pleiotropy** **test** | | |
| --- | --- | --- | --- | --- | --- | --- | --- | --- | --- | --- |
|  |  |  |  |  | **F-statistic** | **Q statistic** | **P** **value** | **Egger** **intercept** | **SE** | **P** **value** |
| ***PCs(IEU)*** | | | | | | | | | |  |
| TL | MV-IVW | 1.287 (1.162-1.413) | 0.064 | 6.65E-06 | 121.621 | 488.310 | 4.25E-44 | 1.003 | 0.002 | 7.61E-02 |
| Alzheimer’s disease |  | 0.891 (0.799-0.984) | 0.047 | 9.60E-02 |  |  |  |  |  |  |
| TL | MVMR Egger | 1.300 (1.175-1.424) | 0.064 | 2.50E-06 |  | 476.324 | 1.88E-42 |  |  |  |
| Alzheimer’s disease |  | 0.956 (0.917-0.995) | 0.020 | 2.77E-02 |  |  |  |  |  |  |
| TL | MVMR Robust | 1.430 (1.284-1.592) | NA | 7.49E-11 |  | NA | |  |  |  |
| Alzheimer’s disease |  | 0.968 (0.946-0.990) | NA | 5.34E-03 |  |  |  |  |  |  |
| TL | MVMR LASSO | 1.343 (1.267-1.419) | 0.039 | 6.94E-19 |  |  |  |  |  |  |
| Alzheimer’s disease |  | 0.997 (0.954-1.039) | 0.022 | 8.71E-02 |  |  |  |  |  |  |
| TL | MVMR Median | 1.337 (1.226-1.447) | 0.056 | 2.34E-09 |  |  |  |  |  |  |
| Alzheimer’s disease |  | 0.987 (0.947-1.027) | 0.020 | 5.13E-01 |  |  |  |  |  |  |
| TL | MV-IVW | 1.252 (1.133-1.372) | 0.061 | 3.55E-05 | 116.272 | 483.212 | 1.09E-40 | 1.004 | 0.002 | 5.25E-02 |
| Liver_iron_content |  | 0.974 (0.876-1.071) | 0.050 | 5.96E-01 |  |  |  |  |  |  |
| TL | MVMR Egger | 1.274 (1.154-1.395) | 0.061 | 8.21E-06 |  | 470.024 | 6.82E-39 |  |  |  |
| Liver_iron_content |  | 0.938 (0.836-1.041) | 0.053 | 2.41E-01 |  |  |  |  |  |  |
| TL | MVMR Robust | 1.378 (1.241-1.528) | NA | 1.52E-09 |  | NA | |  |  |  |
| Liver_iron_content |  | 0.955 (0.909-1.003) | NA | 6.36E-02 |  |  |  |  |  |  |
| TL | MVMR LASSO | 1.286 (1.212-1.360) | 0.038 | 4.01E-14 |  |  |  |  |  |  |
| Liver_iron_content |  | 0.944 (0.792-0.997) | 0.027 | 3.72E-02 |  |  |  |  |  |  |
| TL | MVMR Median | 1.321 (1.210-1.432) | 0.057 | 1.54E-08 |  |  |  |  |  |  |
| Liver_iron_content |  | 0.954 (0.848-1.060) | 0.054 | 3.97E-01 |  |  |  |  |  |  |
| TL | MV-IVW | 1.422 (1.190-1.654) | 0.118 | 3.60E-04 | 37.817 | 107.689 | 3.08E-12 | 1.000 | 0.005 | 9.77E-01 |
| Circulating_leptin_levels |  | 0.886 (0.509-1.263) | 0.193 | 5.54E-01 |  |  |  |  |  |  |
| TL | MVMR Egger | 1.422(1.183-1.661) | 0.122 | 5.42E-04 |  | 107.685 | 1.43E-12 |  |  |  |
| Circulating_leptin_levels |  | 0.891 (0.386-1.395) | 0.257 | 6.71E-01 |  |  |  |  |  |  |
| TL | MVMR Robust | 1.519 (1.275-1.810) | NA | 2.85E-06 |  | NA | |  |  |  |
| Circulating_leptin_levels |  | 0.915 (0.743-1.127) | NA | 4.03E-01 |  |  |  |  |  |  |
| TL | MVMR LASSO | 1.391 (1.224-1.557) | 0.085 | 4.12E-06 |  |  |  |  |  |  |
| Circulating_leptin_levels |  | 0.938 (0.674-1.202) | 0.134 | 6.45E-01 |  |  |  |  |  |  |
| TL | MVMR Median | 1.327 (1.151-1.502) | 0.090 | 2.66E-04 |  |  |  |  |  |  |
| Circulating_leptin_levels |  | 0.870 (0.616-1.124) | 0.130 | 3.14E-01 |  |  |  |  |  |  |
| TL | MV-IVW | 1.259(1.130-1.387) | 0.066 | 8.07E-05 | 35.516 | 1256.300 | 5.93E-98 | 1.000 | 0.002 | 8.67E-01 |
| Sex_hormone_binding_globulin_levels |  | 0.853 (0.690-1.017) | 0.083 | 7.86E-02 |  |  |  |  |  |  |
| TL | MVMR Egger | 1.256 (1.125-1.388) | 0.067 | 1.38E-04 |  | 1256.204 | 1.85E-95 |  |  |  |
| Sex_hormone_binding_globulin_levels |  | 0.871 (0.609-1.132) | 0.134 | 3.33E-01 |  |  |  |  |  |  |
| TL | MVMR Robust | 1.432 (1.289-1.590) | NA | 1.99E-11 |  | NA | |  |  |  |
| Sex_hormone_binding_globulin_levels |  | 1.074 (0.921-1.252) | NA | 3.63E-01 |  |  |  |  |  |  |
| TL | MVMR LASSO | 1.322 (1.240-1.403) | 0.042 | 1.34E-14 |  |  |  |  |  |  |
| Sex_hormone_binding_globulin_levels |  | 1.045 (0.944-1.146) | 0.052 | 3.79E-01 |  |  |  |  |  |  |
| TL | MVMR Median | 1.342 (1.220-1.464) | 0.062 | 4.33E-08 |  |  |  |  |  |  |
| Sex_hormone_binding_globulin_levels |  | 0.929 (0.771-1.086) | 0.080 | 3.76E-01 |  |  |  |  |  |  |
| TL | MV-IVW | 1.264(1.137-1.390) | 0.065 | 4.40E-05 | 88.123 | 504.002 | 4.71E-46 | 1.003 | 0.002 | 1.92E-01 |
| Naive_CD4-CD8-_T_cell %T_cell |  | 1.015 (0.952-1.079) | 0.032 | 6.39E-01 |  |  |  |  |  |  |
| TL | MVMR Egger | 1.259 (1.133-1.385) | 0.064 | 4.96E-05 |  | 497.349 | 5.24E-45 |  |  |  |
| Naive_CD4-CD8-_T_cell %T_cell |  | 0.999 (0.931-1.067) | 0.035 | 9.69E-01 |  |  |  |  |  |  |
| TL | MVMR Robust | 1.388 (1.244-1.548) | NA | 4.67E-09 |  | NA | |  |  |  |
| Naive_CD4-CD8-_T_cell %T_cell |  | 1.006 (0.968-1.046) | NA | 7.46E-01 |  |  |  |  |  |  |
| TL | MVMR LASSO | 1.309 (1.232-1.386) | 0.039 | 2.81E-15 |  |  |  |  |  |  |
| Naive_CD4-CD8-_T_cell %T_cell |  | 1.002 (0.925-1.078) | 0.039 | 9.61E-01 |  |  |  |  |  |  |
| TL | MVMR Median | 1.309 (1.196-1.422) | 0.058 | 8.97E-08 |  |  |  |  |  |  |
| Naive_CD4-CD8-_T_cell %T_cell |  | 1.053 (0.965-1.140) | 0.045 | 2.37E-01 |  |  |  |  |  |  |
| TL | MV-IVW | 1.380 (1.176-1.584) | 0.104 | 2.56E-04 | 16.936 | 203.870 | 2.61E-14 | 0.999 | 0.002 | 5.05E-01 |
| Alzheimer’s disease |  | 0.968 (0.933-1.004) | 0.018 | 8.30E-02 |  |  |  |  |  |  |
| Liver_iron_content |  | 1.009 (0.888-1.131) | 0.062 | 8.81E-01 |  |  |  |  |  |  |
| Circulating_leptin_levels |  | 0.861 (0.532-1.189) | 0.168 | 4.07E-01 |  |  |  |  |  |  |
| Sex_hormone_binding_globulin_levels |  | 0.782 (0.442-1.121) | 0.173 | 2.07E-01 |  |  |  |  |  |  |
| Naive_CD4-CD8-_T_cell %T_cell |  | 0.927 (0.826-1.028) | 0.052 | 1.59E-01 |  |  |  |  |  |  |
| TL | MVMR Egger | 1.246 (0.919-1.572) | 0.167 | 1.40E-01 |  | 202.621 | 2.23E-14 |  |  |  |
| Alzheimer’s disease |  | 0.999 (0.948-1.050) | 0.026 | 9.69E-01 |  |  |  |  |  |  |
| Liver_iron_content |  | 0.999 (0.873-1.125) | 0.064 | 9.88E-01 |  |  |  |  |  |  |
| Circulating_leptin_levels |  | 0.843 (0.509-1.177) | 0.170 | 3.57E-01 |  |  |  |  |  |  |
| Sex_hormone_binding_globulin_levels |  | 0.790 (0.448-1.131) | 0.174 | 2.28E-01 |  |  |  |  |  |  |
| Naive_CD4-CD8-_T_cell %T_cell |  | 0.921 (0.828-1.031) | 0.052 | 1.75E-01 |  |  |  |  |  |  |
| TL | MVMR Robust | 1.471 (1.276-1.697) | NA | 1.13E-07 |  | NA | |  |  |  |
| Alzheimer’s disease |  | 0.971 (0.958-0.010) | NA | 3.75E-05 |  |  |  |  |  |  |
| Liver_iron_content |  | 1.014 (0.964-1.066) | NA | 5.99E-01 |  |  |  |  |  |  |
| Circulating_leptin_levels |  | 0.966 (0.741-1.259) | NA | 7.96E-01 |  |  |  |  |  |  |
| Sex_hormone_binding_globulin_levels |  | 0.700 (0.498-0.984) | NA | 4.00E-02 |  |  |  |  |  |  |
| Naive_CD4-CD8-_T_cell %T_cell |  | 0.904 (0.834-0.979) | NA | 1.35E-02 |  |  |  |  |  |  |
| TL | MVMR LASSO | 1.342 (1.209-1.475) | 0.068 | 2.30E-08 |  |  |  |  |  |  |
| Alzheimer’s disease |  | 0.973 (0.951-0.995) | 0.011 | 1.61E-02 |  |  |  |  |  |  |
| Liver_iron_content |  | 1.023 (0.946-1.099) | 0.039 | 5.61E-01 |  |  |  |  |  |  |
| Circulating_leptin_levels |  | 0.904 (0.681-1.128) | 0.114 | 4.01E-01 |  |  |  |  |  |  |
| Sex_hormone_binding_globulin_levels |  | 0.700 (0.460-0.940) | 0.122 | 1.43E-02 |  |  |  |  |  |  |
| Naive_CD4-CD8-_T_cell %T_cell |  | 0.874 (0.808-0.941) | 0.034 | 2.05E-04 |  |  |  |  |  |  |
| TL | MVMR Median | 1.295 (1.114-1.477) | 0.093 | 9.92E-04 |  |  |  |  |  |  |
| Alzheimer’s disease |  | 0.964 (0.935-0.993) | 0.015 | 1.40E-02 |  |  |  |  |  |  |
| Liver_iron_content |  | 1.006 (0.873-1.140) | 0.068 | 9.26E-01 |  |  |  |  |  |  |
| Circulating_leptin_levels |  | 0.891 (0.604-1.178) | 0.146 | 4.56E-01 |  |  |  |  |  |  |
| Sex_hormone_binding_globulin_levels |  | 0.710 (0.343-1.077) | 0.187 | 1.22E-01 |  |  |  |  |  |  |
| Naive_CD4-CD8-_T_cell %T_cell |  | 0.920 (0.811-1.029) | 0.056 | 1.52E-01 |  |  |  |  |  |  |
| ***PCs(FinnGene)*** | | | | | | | | | | |
| TL | MV-IVW | 1.282 (1.084-1.481) | 0.101 | 5.76E-03 | 119.737 | 205.563 | 3.09E-05 | 1.002 | 0.003 | 5.46E-01 |
| Alzheimer’s disease |  | 0.985 (0.932-1.039) | 0.027 | 5.92E-01 |  |  |  |  |  |  |
| TL | MVMR Egger | 1.290 (1.090-1.491) | 0.102 | 4.57E-03 |  | 204.978 | 1.84E-05 |  |  |  |
| Alzheimer’s disease |  | 0.979 (0.922-1.036) | 0.029 | 4.77E-01 |  |  |  |  |  |  |
| TL | MVMR Robust | 1.523 (1.250-1.857) | NA | 3.10E-05 |  | NA | |  |  |  |
| Alzheimer’s disease |  | 0.984 (0.960-1.008) | NA | 1.85E-01 |  |  |  |  |  |  |
| TL | MVMR LASSO | 1.415 (1.243-1.587) | 0.088 | 2.33E-06 |  |  |  |  |  |  |
| Alzheimer’s disease |  | 0.981 (0.939-1.023) | 0.022 | 3.82E-01 |  |  |  |  |  |  |
| TL | MVMR Median | 1.329 (1.076-1.583) | 0.129 | 1.09E-02 |  |  |  |  |  |  |
| Alzheimer’s disease |  | 0.978 (0.926-1.029) | 0.026 | 3.98E-01 |  |  |  |  |  |  |
| TL | MV-IVW | 1.292 (1.090-1.495) | 0.103 | 4.65E-03 | 118.605 | 210.578 | 7.63E-06 | 1.004 | 0.003 | 2.60E-01 |
| Liver_iron_content |  | 1.010 (0.816-1.204) | 0.099 | 9.21E-01 |  |  |  |  |  |  |
| TL | MVMR Egger | 1.314 (1.108-1.519) | 0.105 | 2.78E-03 |  | 208.513 | 8.99E-06 |  |  |  |
| Liver_iron_content |  | 0.965 (0.756-1.174) | 0.107 | 7.41E-01 |  |  |  |  |  |  |
| TL | MVMR Robust | 1.541 (1.260-1.886) | NA | 2.65E-05 |  | NA | |  |  |  |
| Liver_iron_content |  | 0.995 (0.874-1.134) | NA | 9.42E-01 |  |  |  |  |  |  |
| TL | MVMR LASSO | 1.426 (1.251-1.602) | 0.089 | 1.87E-06 |  |  |  |  |  |  |
| Liver_iron_content |  | 0.975 (0.821-1.129) | 0.079 | 7.53E-01 |  |  |  |  |  |  |
| TL | MVMR Median | 0.666 (0.376-0.957) | 0.148 | 5.14E-02 |  |  |  |  |  |  |
| Liver_iron_content |  | 0.954 (0.848-1.060) | 0.134 | 2.45E-02 |  |  |  |  |  |  |
| TL | MV-IVW | 1.331(0.980-1.683) | 0.179 | 6.46E-02 | 37.817 | 37.756 | 4.89E-02 | 0.995 | 0.008 | 4.98E-01 |
| Circulating_leptin_levels |  | 0.989 (0.407-1.571) | 0.297 | 9.71E-01 |  |  |  |  |  |  |
| TL | MVMR Egger | 1.316 (0.959-1.674) | 0.183 | 8.31E-02 |  | 37.047 | 4.33E-02 |  |  |  |
| Circulating_leptin_levels |  | 1.159 (0.393-1.925) | 0.391 | 6.85E-01 |  |  |  |  |  |  |
| TL | MVMR Robust | 1.404 (1.039-1.898) | NA | 2.72E-02 |  | NA | |  |  |  |
| Circulating_leptin_levels |  | 1.000 (0.551-1.814) | NA | 1.00E+00 |  |  |  |  |  |  |
| TL | MVMR LASSO | 1.405 (1.107-1.702) | 0.152 | 7.78E-03 |  |  |  |  |  |  |
| Circulating_leptin_levels |  | 1.031 (0.548-1.514) | 0.247 | 9.01E-01 |  |  |  |  |  |  |
| TL | MVMR Median | 1.356 (0.927-1.785) | 0.219 | 1.04E-01 |  |  |  |  |  |  |
| Circulating_leptin_levels |  | 1.341 (0.557-2.225) | 0.400 | 3.94E-01 |  |  |  |  |  |  |
| TL | MV-IVW | 1.207 (1.010-1.403) | 0.100 | 3.94E-02 | 35.892 | 450.935 | 3.65E-04 | 1.002 | 0.002 | 4.18E-01 |
| Sex_hormone_binding_globulin_levels |  | 0.721 (0.476-0.966) | 0.125 | 2.52E-02 |  |  |  |  |  |  |
| TL | MVMR Egger | 1.224 (1.023-1.425) | 0.103 | 2.91E-02 |  | 450.037 | 3.53E-04 |  |  |  |
| Sex_hormone_binding_globulin_levels |  | 0.599 (0.216-0.981) | 0.195 | 3.99E-02 |  |  |  |  |  |  |
| TL | MVMR Robust | 1.477 (1.197-1.823) | NA | 2.80E-04 |  | NA | |  |  |  |
| Sex_hormone_binding_globulin_levels |  | 0.794 (0.589-1.069) | NA | 1.28E-01 |  |  |  |  |  |  |
| TL | MVMR LASSO | 1.399 (1.210-1.588) | 0.097 | 3.62E-05 |  |  |  |  |  |  |
| Sex_hormone_binding_globulin_levels |  | 0.724 (0.505-0.943) | 0.112 | 1.37E-02 |  |  |  |  |  |  |
| TL | MVMR Median | 1.178 (0.901-1.455) | 0.141 | 2.08E-01 |  |  |  |  |  |  |
| Sex_hormone_binding_globulin_levels |  | 0.985 (0.629-1.341) | 0.182 | 9.34E-01 |  |  |  |  |  |  |
| TL | MV-IVW | 1.244 (1.047-1.442) | 0.101 | 1.54E-02 | 87.889 | 196.116 | 8.07E-05 | 1.003 | 0.003 | 2.66E-01 |
| Naive_CD4-CD8-_T_cell %T_cell |  | 1.091 (1.008-1.174) | 0.042 | 3.11E-02 |  |  |  |  |  |  |
| TL | MVMR Egger | 1.237 (1.040-1.435) | 0.101 | 1.86E-02 |  | 194.209 | 9.16E-05 |  |  |  |
| Naive_CD4-CD8-_T_cell %T_cell |  | 1.076 (0.989-1.163) | 0.044 | 8.78E-02 |  |  |  |  |  |  |
| TL | MVMR Robust | 1.464 (1.207-1.776) | NA | 1.11E-04 |  | NA | |  |  |  |
| Naive_CD4-CD8-_T_cell %T_cell |  | 1.087 (1.045-1.130) | NA | 2.92E-05 |  |  |  |  |  |  |
| TL | MVMR LASSO | 1.352 (1.180-1.523) | 0.088 | 6.14E-05 |  |  |  |  |  |  |
| Naive_CD4-CD8-_T_cell %T_cell |  | 1.077 (1.010-1.144) | 0.034 | 2.47E-02 |  |  |  |  |  |  |
| TL | MVMR Median | 1.272 (1.015-1.529) | 0.131 | 3.77E-02 |  |  |  |  |  |  |
| Naive_CD4-CD8-_T_cell %T_cell |  | 1.120 (0.969-1.272) | 0.077 | 1.19E-01 |  |  |  |  |  |  |
| TL | MV-IVW | 1.378 (1.076-1.680) | 0.154 | 1.42E-02 | 17.064 | 46.538 | 9.93E-01 | 0.997 | 0.003 | 4.08E-01 |
| Alzheimer’s disease |  | 0.999 (0.978-1.020) | 0.011 | 9.23E-01 |  |  |  |  |  |  |
| Liver_iron_content |  | 1.153 (1.030-1.275) | 0.063 | 1.48E-02 |  |  |  |  |  |  |
| Circulating_leptin_levels |  | 0.950 (0.426-1.474) | 0.268 | 2.87E-01 |  |  |  |  |  |  |
| Sex_hormone_binding_globulin_levels |  | 1.053 (0.196-1.910) | 0.437 | 9.03E-01 |  |  |  |  |  |  |
| Naive_CD4-CD8-_T_cell %T_cell |  | 0.835 (0.662-1.009) | 0.089 | 6.28E-02 |  |  |  |  |  |  |
| TL | MVMR Egger | 1.246 (0.919-1.572) | 0.167 | 1.40E-01 |  | 74.948 | 3.83E-01 |  |  |  |
| Alzheimer’s disease |  | 0.999 (0.948-1.050) | 0.026 | 9.69E-01 |  |  |  |  |  |  |
| Liver_iron_content |  | 1.155 (0.941-1.369) | 0.109 | 1.55E-02 |  |  |  |  |  |  |
| Circulating_leptin_levels |  | 0.918 (0.387-1.449) | 0.271 | 7.61E-01 |  |  |  |  |  |  |
| Sex_hormone_binding_globulin_levels |  | 0.798 (0.297-1.299) | 0.256 | 4.30E-01 |  |  |  |  |  |  |
| Naive_CD4-CD8-_T_cell %T_cell |  | 0.898 (0.743-1.053) | 0.079 | 1.96E-01 |  |  |  |  |  |  |
| TL | MVMR Robust | 1.459 (1.079-1.973) | NA | 1.42E-02 |  | NA | |  |  |  |
| Alzheimer’s disease |  | 0.999 (0.978-1.021) | NA | 9.23E-01 |  |  |  |  |  |  |
| Liver_iron_content |  | 1.165 (1.030-1.317) | NA | 1.48E-02 |  |  |  |  |  |  |
| Circulating_leptin_levels |  | 1.580 (0.681-3.664) | NA | 2.87E-01 |  |  |  |  |  |  |
| Sex_hormone_binding_globulin_levels |  | 1.054 (0.448-2.484) | NA | 9.03E-01 |  |  |  |  |  |  |
| Naive_CD4-CD8-_T_cell %T_cell |  | 0.848 (0.713-1.009) | NA | 6.28E-02 |  |  |  |  |  |  |
| TL | MVMR LASSO | 1.405 (1.084-1.726) | 0.164 | 1.34E-02 |  |  |  |  |  |  |
| Alzheimer’s disease |  | 0.994 (0.944-1.044) | 0.025 | 8.12E-01 |  |  |  |  |  |  |
| Liver_iron_content |  | 1.143 (0.938-1.348) | 0.105 | 1.71E-01 |  |  |  |  |  |  |
| Circulating_leptin_levels |  | 1.489 (0.906-2.072) | 0.297 | 1.00E-02 |  |  |  |  |  |  |
| Sex_hormone_binding_globulin_levels |  | 0.975 (0.438-1.511) | 0.274 | 9.27E-01 |  |  |  |  |  |  |
| Naive_CD4-CD8-_T_cell %T_cell |  | 0.840 (0.683-0.997) | 0.080 | 4.54E-02 |  |  |  |  |  |  |
| TL | MVMR Median | 1.174 (0.742-1.607) | 0.221 | 4.29E-01 |  |  |  |  |  |  |
| Alzheimer’s disease |  | 0.999 (0.944-1.055) | 0.028 | 9.84E-01 |  |  |  |  |  |  |
| Liver_iron_content |  | 1.183 (0.864-1.503) | 0.163 | 2.61E-01 |  |  |  |  |  |  |
| Circulating_leptin_levels |  | 0.954 (0.263-1.646) | 0.353 | 8.97E-01 |  |  |  |  |  |  |
| Sex_hormone_binding_globulin_levels |  | 0.798 (0.483-1.645) | 0.432 | 6.41E-01 |  |  |  |  |  |  |
| Naive_CD4-CD8-_T_cell %T_cell |  | 0.891 (0.675-1.107) | 0.110 | 3.22E-01 |  |  |  |  |  |  |

^†^β (95% CI) represents the associations of each SD increase/decrease in TL with PCs.

Abbreviations: TL=Telomere Length; BMR=Basal metabolic rate; CI=confidence interval; MV-IVW=multivariable inverse variance weighted; MVMR Egger=multivariable Mendelian Randomization Egger; PCs=prostate cancers; OR=odds TLtio; SD=standard deviation.

**Table S15. Selected mediators of the impact of LTL on PCs and the rationale for selection based on published literature**

| **Potential mediators** | **Conclusions** | **PMID** |
| --- | --- | --- |
| Liver_iron_content | Genetically predicted increase in iron status was associated with decreased risk of PC. | 36561528 |
| Body_fat_percentage | Risk of PC-related death is increased in men with higher total and central adiposity. | 35509091 |
| Sex_hormone_binding_globulin_levels | High sex hormone binding globulin levels were associated with a 3.2-fold risk of detection of PC on prostate biopsy. | 23181478 |
| Circulating_leptin_levels | Men with high-volume PC exhibited higher leptin concentrations. | 11170133 |
| Mean_corpuscular_hemoglobin | Higher mean corpuscular hemoglobin were related to a lower risk of PC. | 32457180 |
| Arm_fat_percentage  (right / left) | Men with obesity tended to be diagnosed with more advanced PC. Body fat percentage exhibited strong association with advanced PC. | 25264293 |
| Total_fatty_acids | Intake of dietary fat, scaled by the total caloric intake, was associated with increased risk of PC. | 30385837 |
| Polyunsaturated_fatty_acids | Decreased risks were observed for short-chain PUFAs, whereas increased risks were observed for long-chain PUFAs among men <62 years. | 27490808 |
| Saturated_fatty_acids | Increased risk of PC was associated with intake of saturated fatty acid. | 30385837 |
| Vitamin_D | Higher 25-hydroxyvitamin D levels were associated with a 57% decrease in the risk of lethal PC. | 22499501 |
| Vitamin_E | Vitamin E supplementation significantly increased the risk of PC among healthy men. | 21990298 |
| Fresh_tomato_intake | Intake of lycopene or other compounds in fresh tomatoes may reduce the risk of PC. | 7473833 |
| Naive_CD4-CD8-_T_cell %T_cell | Double negative CAR-T cells targeting CD19 (CD19-CAR-DNT) can effectively eradicate hematomas and solid tumors without causing graft-versus-host disease. | 35452255 |
| Epstein_Barr_virus_antibody_levels | Patients diagnosed with infectious mononucleosis (indicative of Epstein-Barr virus infection) had a strong tendency to positively correlate with PC. | 36497319 |
| Coffee_intake | Coffee consumption may be negatively correlated with the risk of fatal PC. | 24276028 |
| Alcoholic_drinks_per_week | A positive association between alcohol consumption and risk of PC was indicated. | 25422909 |
| Lifetime_number_of_sexual_partners | An increase of 10 female sexual partners was associated with a 1.10-fold increase of PC risk. | 30122473 |
| Cigarettes_smoked_per_day | A statistically significant association between cigarette smoking and fatal PC was observed. | 25242554 |
| Fried_potatoes_intake | Higher intake of fried food was correlated with a 35% increased risk of PC. | 26114920 |
| Hypertension | Hypertension is indicated to be associated with an increased risk of PC. | 27511796 |
| Alzheimer's_disease | Patients with AD might have a lower chance of developing PC. | 34482613 |
| Coronary_heart_disease | Multivariate analysis showed an association of CAD with a 35% increased risk of PC diagnosis. | 22315364 |

Abbreviations: PUFA=Polyunsaturated fatty acids; AD=Alzheimer's disease; CAD=Coronary heart disease; PC=Prostate cancer.

**Table S16. Univariable MR estimates for the causal effect between nutrition factors and five causal mediators of PCs**

| **Exposure** | **Outcome** | **No. of SNP** | **^†^β (95% CI)**^†^ | **P value** | **FDR q-value** | **F-statistic** |
| --- | --- | --- | --- | --- | --- | --- |
| Total_fatty_acids | Liver_iron_content | 62 | 0.942 (0.883-1.006) | 7.57E-02 | 2.85E-01 | 120.190 |
|  | Sex_hormone_binding_globulin_levels | 62 | 0.961 (0.913-1.012) | 1.30E-01 | 4.36E-01 | 120.190 |
|  | Circulating_leptin_levels | 21 | 0.934 (0.843-1.035) | 1.92E-01 | 5.93E-01 | 175.704 |
|  | Naive_CD4-CD8-_T_cell %T_cell | 56 | 1.035 (0.893-1.201) | 6.46E-01 | 8.96E-01 | 125.436 |
|  | Alzheimer's_disease | 60 | 0.917 (0.801-1.050) | 2.12E-01 | 6.04E-01 | 121.133 |
| Saturated_fatty_acids | Liver_iron_content | 52 | 0.928 (0.863-0.997) | 4.09E-02 | 2.02E-01 | 117.717 |
|  | Sex_hormone_binding_globulin_levels | 52 | 0.945 (0.890-1.004) | 6.69E-02 | 2.85E-01 | 117.717 |
|  | Circulating_leptin_levels | 17 | 0.904 (0.795-1.029) | 1.28E-01 | 4.36E-01 | 162.814 |
|  | Naive_CD4-CD8-_T_cell %T_cell | 50 | 1.060 (0.895-1.255) | 4.99E-01 | 8.44E-01 | 119.751 |
|  | Alzheimer's_disease | 50 | 0.901 (0.769-1.056) | 1.97E-01 | 5.93E-01 | 119.867 |
| Polyunsaturated_fatty_acids | Liver_iron_content | 64 | 0.957 (0.904-1.013) | 1.28E-01 | 4.36E-01 | 129.751 |
|  | Sex_hormone_binding_globulin_levels | 64 | 0.995 (0.956-1.036) | 8.15E-01 | 9.62E-01 | 129.751 |
|  | Circulating_leptin_levels | 23 | 0.971 (0.914-1.031) | 3.38E-01 | 7.56E-01 | 170.993 |
|  | Naive_CD4-CD8-_T_cell %T_cell | 58 | 0.982 (0.863-1.116) | 7.75E-01 | 9.62E-01 | 138.065 |
|  | Alzheimer's_disease | 57 | 0.886 (0.623-1.259) | 4.98E-01 | 8.44E-01 | 131.451 |
| Vitamin_D | NA | | | | | |
| Vitamin_E | NA | | | | | |
| Fresh_tomato_intake | NA | | | | | |
| Coffee_intake | Liver_iron_content | 39 | 0.874 (0.696-1.098) | 2.48E-01 | 6.66E-01 | 72.712 |
|  | Sex_hormone_binding_globulin_levels | 39 | 0.998 (0.862-1.156) | 9.80E-01 | 9.90E-01 | 72.712 |
|  | Circulating_leptin_levels | 9 | 1.582 (0.800-3.129) | 1.87E-01 | 5.93E-01 | 185.779 |
|  | Naive_CD4-CD8-_T_cell %T_cell | 39 | 1.132 (0.654-1.960) | 6.58E-01 | 8.96E-01 | 72.712 |
|  | Alzheimer's_disease | 38 | 1.245 (0.727-2.131) | 4.24E-01 | 8.15E-01 | 72.712 |
| Alcoholic_drinks_per_week | Liver_iron_content | 33 | 1.203 (0.983-1.471) | 7.33E-02 | 2.85E-01 | 77.308 |
|  | Sex_hormone_binding_globulin_levels | 34 | 1.022 (0.869-1.203) | 7.89E-01 | 9.62E-01 | 77.308 |
|  | Circulating_leptin_levels | 7 | 0.969 (0.487-1.928) | 9.28E-01 | 9.67E-01 | 194.470 |
|  | Naive_CD4-CD8-_T_cell %T_cell | 34 | 1.192 (0.816-1.740) | 3.64E-01 | 7.70E-01 | 77.308 |
|  | Alzheimer's_disease | 34 | 1.364 (0.746-2.492) | 3.14E-01 | 7.22E-01 | 77.308 |
| Lifetime_number_of_sexual_partners | Liver_iron_content | 61 | 1.120 (0.933-1.344) | 2.24E-01 | 6.19E-01 | 38.288 |
|  | Sex_hormone_binding_globulin_levels | 61 | 1.045 (0.996-1.097) | 7.50E-02 | 2.85E-01 | 38.288 |
|  | Circulating_leptin_levels | 7 | 0.562 (0.347-0.912) | 1.96E-02 | 1.08E-01 | 39.260 |
|  | Naive_CD4-CD8-_T_cell %T_cell | 60 | 0.882 (0.528-1.472) | 6.31E-01 | 8.96E-01 | 38.288 |
|  | Alzheimer's_disease | 60 | 1.359 (0.706-2.619) | 3.59E-01 | 7.70E-01 | 38.288 |
| Cigarettes_smoked_per_day | Liver_iron_content | 22 | 1.009 (0.713-1.428) | 6.13E-01 | 8.96E-01 | 100.147 |
|  | Sex_hormone_binding_globulin_levels | 22 | 0.997 (0.793-1.253) | 3.00E-01 | 7.22E-01 | 100.147 |
|  | Circulating_leptin_levels | 2 | 0.917 (0.754-1.115) | 8.19E-01 | 9.62E-01 | 496.897 |
|  | Naive_CD4-CD8-_T_cell %T_cell | 22 | 0.711 (0.441-1.145) | 8.45E-01 | 9.62E-01 | 100.147 |
|  | Alzheimer's_disease | 22 | 0.982 (0.784-1.228) | 3.84E-01 | 7.70E-01 | 100.147 |
| Fried_potatoes_intake | Liver_iron_content | 1 | 0.902 (0.589-1.379) | 6.33E-01 | 8.96E-01 | 33.362 |
|  | Sex_hormone_binding_globulin_levels | 1 | 1.020 (0.969-1.074) | 4.50E-01 | 8.42E-01 | 33.362 |
|  | Circulating_leptin_levels | NA | | | | |
|  | Naive_CD4-CD8-_T_cell %T_cell | 1 | 1.428 (0.363-5.617) | 6.10E-01 | 8.96E-01 | 33.362 |
|  | Alzheimer's_disease | 1 | 0.829 (0.234-2.940) | 7.72E-01 | 9.62E-01 | 33.362 |
| Liver_iron_content | Total_fatty_acids | 10 | 0.997 (0.952-1.045) | 9.12E-01 | 9.63E-01 | 230.653 |
|  | Polyunsaturated_fatty_acids | 10 | 0.980 (0.935-1.026) | 3.85E-01 | 7.70E-01 | 230.653 |
|  | Saturated_fatty_acids | 10 | 0.997 (0.955-1.040) | 8.85E-01 | 9.63E-01 | 230.653 |
|  | Vitamin_D | 8 | 0.999 (0.997-1.001) | 4.75E-01 | 8.44E-01 | 268.849 |
|  | Vitamin_E | 8 | 0.984 (0.954-1.015) | 3.15E-01 | 7.22E-01 | 268.849 |
|  | Fresh_tomato_intake | 8 | 1.014 (0.969-1.061) | 5.61E-01 | 8.93E-01 | 268.849 |
|  | Coffee_intake | 8 | 0.995 (0.981-1.008) | 4.25E-01 | 8.15E-01 | 268.849 |
|  | Alcoholic_drinks_per_week | 7 | 0.997 (0.985-1.010) | 6.42E-01 | 8.96E-01 | 262.015 |
|  | Lifetime_number_of_sexual_partners | 8 | 1.003 (0.993-1.014) | 5.12E-01 | 8.44E-01 | 268.849 |
|  | Cigarettes_smoked_per_day | 7 | 0.988 (0.955-1.022) | 4.93E-01 | 8.44E-01 | 262.015 |
|  | Fried_potatoes_intake | 8 | 1.004 (0.961-1.048) | 8.70E-01 | 9.62E-01 | 268.849 |
| Sex_hormone_binding_globulin_levels | Total_fatty_acids | 386 | 0.740 (0.662-0.827) | 1.01E-07 | 2.37E-06 | 118.827 |
|  | Polyunsaturated_fatty_acids | 386 | 0.965 (0.862-1.081) | 5.41E-01 | 8.77E-01 | 118.827 |
|  | Saturated_fatty_acids | 386 | 0.713 (0.641-0.792) | 3.10E-10 | 2.91E-08 | 118.827 |
|  | Vitamin_D | 344 | 1.007 (1.002-1.012) | 2.75E-03 | 3.14E-02 | 117.402 |
|  | Vitamin_E | 351 | 0.992 (0.929-1.059) | 8.14E-01 | 9.62E-01 | 117.557 |
|  | Fresh_tomato_intake | 351 | 0.991 (0.908-1.081) | 8.40E-01 | 9.62E-01 | 117.557 |
|  | Coffee_intake | 351 | 1.047 (1.015-1.080) | 3.56E-03 | 3.35E-02 | 117.557 |
|  | Alcoholic_drinks_per_week | 346 | 1.047 (1.012-1.083) | 8.63E-03 | 6.76E-02 | 117.581 |
|  | Lifetime_number_of_sexual_partners | 351 | 1.014 (0.987-1.042) | 3.14E-01 | 7.22E-01 | 117.557 |
|  | Cigarettes_smoked_per_day | 346 | 0.903 (0.835-0.976) | 1.03E-02 | 6.92E-02 | 117.581 |
|  | Fried_potatoes_intake | 351 | 0.977 (0.913-1.046) | 5.03E-01 | 8.44E-01 | 117.557 |
| Circulating_leptin_levels | Total_fatty_acids | 3 | 0.762 (0.602-0.965) | 2.41E-02 | 1.26E-01 | 56.186 |
|  | Polyunsaturated_fatty_acids |  | 0.870 (0.788-0.962) | 6.36E-03 | 5.43E-02 |  |
|  | Saturated_fatty_acids |  | 0.741 (0.585-0.939) | 1.30E-02 | 8.15E-02 |  |
|  | Vitamin_D |  | 1.000 (0.983-1.017) | 9.72E-01 | 9.90E-01 |  |
|  | Vitamin_E |  | 1.122 (0.828-1.520) | 4.57E-01 | 8.42E-01 |  |
|  | Fresh_tomato_intake |  | 0.967 (0.807-1.158) | 7.14E-01 | 9.59E-01 |  |
|  | Coffee_intake |  | 1.187 (0.987-1.429) | 6.93E-02 | 2.85E-01 |  |
|  | Alcoholic_drinks_per_week |  | 0.979 (0.830-1.154) | 7.98E-01 | 9.62E-01 |  |
|  | Lifetime_number_of_sexual_partners |  | 0.943 (0.827-1.076) | 3.84E-01 | 7.70E-01 |  |
|  | Cigarettes_smoked_per_day |  | 0.858 (0.757-0.971) | 1.52E-02 | 8.93E-02 |  |
|  | Fried_potatoes_intake |  | 1.011 (0.768-1.332) | 9.36E-01 | 9.67E-01 |  |
| Naive_CD4-CD8-_T_cell %T_cell | Total_fatty_acids | 1 | 0.840 (0.791-0.891) | 9.26E-09 | 2.90E-07 | 33.960 |
|  | Polyunsaturated_fatty_acids |  | 0.840 (0.792-0.891) | 5.44E-09 | 2.56E-07 |  |
|  | Saturated_fatty_acids |  | 0.861 (0.811-0.914) | 7.87E-07 | 1.48E-05 |  |
|  | Vitamin_D |  | 1.000 (0.994-1.005) | 8.98E-01 | 9.63E-01 |  |
|  | Vitamin_E |  | 0.991 (0.915-1.073) | 8.18E-01 | 9.62E-01 |  |
|  | Fresh_tomato_intake |  | 1.027 (0.914-1.153) | 6.57E-01 | 8.96E-01 |  |
|  | Coffee_intake |  | 1.015 (0.992-1.039) | 2.02E-01 | 5.93E-01 |  |
|  | Alcoholic_drinks_per_week |  | 1.044 (1.015-1.073) | 3.01E-03 | 3.14E-02 |  |
|  | Lifetime_number_of_sexual_partners |  | 0.992 (0.966-1.019) | 5.70E-01 | 8.93E-01 |  |
|  | Cigarettes_smoked_per_day |  | 0.955 (0.880-1.036) | 2.65E-01 | 6.73E-01 |  |
|  | Fried_potatoes_intake |  | 0.992 (0.907-1.085) | 8.54E-01 | 9.62E-01 |  |
| Alzheimer's_disease | Total_fatty_acids | 6 | 1.058 (1.023-1.094) | 1.11E-03 | 1.49E-02 | 265.120 |
|  | Polyunsaturated_fatty_acids | 6 | 1.070 (1.016-1.126) | 9.86E-03 | 6.92E-02 | 265.120 |
|  | Saturated_fatty_acids | 6 | 1.044 (1.018-1.070) | 6.65E-04 | 1.04E-02 | 265.120 |
|  | Vitamin_D | 5 | 0.999 (0.985-1.013) | 8.61E-01 | 9.62E-01 | 363.117 |
|  | Vitamin_E | 5 | 0.999 (0.985-1.013) | 8.61E-01 | 9.62E-01 | 299.901 |
|  | Fresh_tomato_intake | 5 | 1.002 (0.983-1.021) | 8.29E-01 | 9.62E-01 | 299.901 |
|  | Coffee_intake | 6 | 1.000 (0.995-1.004) | 9.02E-01 | 9.63E-01 | 265.120 |
|  | Alcoholic_drinks_per_week | 6 | 1.000 (0.996-1.004) | 9.90E-01 | 9.90E-01 | 265.120 |
|  | Lifetime_number_of_sexual_partners | 6 | 1.004 (1.000-1.009) | 6.79E-02 | 2.85E-01 | 265.120 |
|  | Cigarettes_smoked_per_day | 6 | 0.987 (0.965-1.010) | 2.65E-01 | 6.73E-01 | 265.120 |
|  | Fried_potatoes_intake | 5 | 0.995 (0.979-1.013) | 5.98E-01 | 8.96E-01 | 299.901 |

^†^β (95% CI) represents the associations of each SD increase/decrease in each potential mediator with TL.

Abbreviations: CI=confidence interval; FDR=false discovery rate; MR=Mendelian Randomization; NA=not applicable; No=number; SNP=single nucleotide polymorphism.

**Table S17. Univariable MR pleiotropy and heterogeneity test for the associations between nutrition, habits and customs factors with five causal mediators of PCs**

| **Horizontal pleiotropy test** | | | | | |
| --- | --- | --- | --- | --- | --- |
| **Exposure** | **Outcome** | | **Egger_intercept** | **Intercept_se** | **Pintercept** |
| Total_fatty_acids | Liver_iron_content | | -2.80E-03 | 3.19E-03 | 3.84E-01 |
|  | Sex_hormone_binding_globulin_levels | | -9.41E-04 | 2.50E-03 | 7.08E-01 |
|  | Circulating_leptin_levels | | -5.07E-03 | 6.78E-03 | 4.64E-01 |
|  | Naive_CD4-CD8-_T_cell %T_cell | | -3.35E-03 | 7.36E-03 | 6.51E-01 |
|  | Alzheimer's_disease | | 5.71E-03 | 6.57E-03 | 3.89E-01 |
| Saturated_fatty_acids | Liver_iron_content | | -6.17E-03 | 3.40E-03 | 7.59E-02 |
|  | Sex_hormone_binding_globulin_levels | | -2.35E-03 | 2.91E-03 | 4.23E-01 |
|  | Circulating_leptin_levels | | -1.96E-03 | 8.64E-03 | 8.24E-01 |
|  | Naive_CD4-CD8-_T_cell %T_cell | | -2.07E-03 | 8.19E-03 | 8.01E-01 |
|  | Alzheimer's_disease | | 4.31E-03 | 7.70E-03 | 5.78E-01 |
| Polyunsaturated_fatty_acids | Liver_iron_content | | 1.21E-03 | 3.26E-03 | 7.12E-01 |
|  | Sex_hormone_binding_globulin_levels | | 1.24E-03 | 2.31E-03 | 5.92E-01 |
|  | Circulating_leptin_levels | | -8.14E-04 | 4.27E-03 | 8.51E-01 |
|  | Naive_CD4-CD8-_T_cell %T_cell | | 1.65E-03 | 7.48E-03 | 8.27E-01 |
|  | Alzheimer's_disease | | -3.51E-02 | 2.05E-02 | 9.26E-02 |
| Vitamin_D | NA | | | | |
| Vitamin_E |  |  |  |  |  |
| Fresh_tomato_intake |  |  |  |  |  |
| Coffee_intake | Liver_iron_content | | -9.60E-04 | 3.84E-03 | 8.04E-01 |
|  | Sex_hormone_binding_globulin_levels | | 2.13E-03 | 2.46E-03 | 3.93E-01 |
|  | Circulating_leptin_levels | | 2.48E-02 | 1.54E-02 | 1.52E-01 |
|  | Naive_CD4-CD8-_T_cell %T_cell | | -2.70E-03 | 9.35E-03 | 7.74E-01 |
|  | Alzheimer's_disease | | -7.03E-04 | 9.17E-03 | 9.39E-01 |
| Alcoholic_drinks_per_week | Liver_iron_content | | -2.40E-03 | 3.46E-03 | 4.94E-01 |
|  | Sex_hormone_binding_globulin_levels | | 2.01E-03 | 2.78E-03 | 4.75E-01 |
|  | Circulating_leptin_levels | | 4.50E-03 | 1.58E-02 | 7.88E-01 |
|  | Naive_CD4-CD8-_T_cell %T_cell | | 7.08E-04 | 6.62E-03 | 9.15E-01 |
|  | Alzheimer's_disease | | 2.70E-03 | 1.22E-02 | 8.26E-01 |
| Lifetime_number_of_sexual_partners | Liver_iron_content | | 4.00E-05 | 5.59E-03 | 9.94E-01 |
|  | Sex_hormone_binding_globulin_levels | | 1.65E-03 | 1.48E-03 | 2.69E-01 |
|  | Circulating_leptin_levels | | 1.47E-02 | 2.46E-02 | 5.77E-01 |
|  | Naive_CD4-CD8-_T_cell %T_cell | | 7.81E-03 | 1.65E-02 | 6.38E-01 |
|  | Alzheimer's_disease | | 2.15E-02 | 2.01E-02 | 2.88E-01 |
| Cigarettes_smoked_per_day | Liver_iron_content | | 6.03E-03 | 5.98E-03 | 3.25E-01 |
|  | Sex_hormone_binding_globulin_levels | | -1.81E-03 | 1.03E-03 | 9.56E-02 |
|  | Circulating_leptin_levels | | NA | | |
|  | Naive_CD4-CD8-_T_cell %T_cell | | -1.91E-02 | 9.15E-03 | 4.96E-02 |
|  | Alzheimer's_disease | | -7.73E-03 | 1.18E-02 | 5.19E-01 |
| Fried_potatoes_intake | NA | | | | |
| Liver_iron_content | Total_fatty_acids | | 4.57E-03 | 5.62E-03 | 4.40E-01 |
|  | Polyunsaturated_fatty_acids | | 5.67E-03 | 5.45E-03 | 3.29E-01 |
|  | Saturated_fatty_acids | | 3.31E-03 | 5.18E-03 | 5.40E-01 |
|  | Vitamin_D | | 5.91E-05 | 3.02E-04 | 8.51E-01 |
|  | Vitamin_E | | 1.89E-03 | 3.91E-03 | 6.46E-01 |
|  | Fresh_tomato_intake | | -4.64E-03 | 5.72E-03 | 4.48E-01 |
|  | Coffee_intake | | -8.02E-05 | 1.83E-03 | 9.66E-01 |
|  | Alcoholic_drinks_per_week | | -9.82E-04 | 1.53E-03 | 5.49E-01 |
|  | Lifetime_number_of_sexual_partners | | 1.81E-03 | 1.31E-03 | 2.15E-01 |
|  | Cigarettes_smoked_per_day | | -5.47E-03 | 4.00E-03 | 2.30E-01 |
|  | Fried_potatoes_intake | | -3.09E-03 | 5.71E-03 | 6.08E-01 |
| Sex_hormone_binding_globulin_levels | Total_fatty_acids | | 1.11E-03 | 1.26E-03 | 3.78E-01 |
|  | Polyunsaturated_fatty_acids | | 2.95E-03 | 1.27E-03 | 2.10E-02 |
|  | Saturated_fatty_acids | | 8.06E-04 | 1.20E-03 | 5.01E-01 |
|  | Vitamin_D | | 8.67E-05 | 5.25E-05 | 9.95E-02 |
|  | Vitamin_E | | -1.38E-03 | 7.45E-04 | 6.57E-02 |
|  | Fresh_tomato_intake | | -2.27E-03 | 9.89E-04 | 2.25E-02 |
|  | Coffee_intake | | -5.55E-04 | 3.51E-04 | 1.14E-01 |
|  | Alcoholic_drinks_per_week | | 4.11E-04 | 3.91E-04 | 2.94E-01 |
|  | Lifetime_number_of_sexual_partners | | 6.48E-04 | 3.06E-04 | 3.49E-02 |
|  | Cigarettes_smoked_per_day | | -1.42E-03 | 8.87E-04 | 1.09E-01 |
|  | Fried_potatoes_intake | | -1.25E-04 | 7.78E-04 | 8.72E-01 |
| Circulating_leptin_levels | Total_fatty_acids | | 3.41E-03 | 6.85E-02 | 9.68E-01 |
|  | Polyunsaturated_fatty_acids | | 1.03E-02 | 2.71E-02 | 7.68E-01 |
|  | Saturated_fatty_acids | | -5.66E-03 | 6.84E-02 | 9.47E-01 |
|  | Vitamin_D | | 1.41E-03 | 4.64E-03 | 8.12E-01 |
|  | Vitamin_E | | -1.20E-02 | 8.75E-02 | 9.13E-01 |
|  | Fresh_tomato_intake | | -7.40E-03 | 3.72E-02 | 8.75E-01 |
|  | Coffee_intake | | -3.85E-02 | 3.76E-02 | 4.93E-01 |
|  | Alcoholic_drinks_per_week | | 2.31E-02 | 4.19E-02 | 6.79E-01 |
|  | Lifetime_number_of_sexual_partners | | 1.79E-02 | 3.36E-02 | 6.89E-01 |
|  | Cigarettes_smoked_per_day | | 1.48E-02 | 2.55E-02 | 6.65E-01 |
|  | Fried_potatoes_intake | | 7.97E-02 | 2.86E-02 | 2.19E-01 |
| Naive_CD4-CD8-_T_cell %T_cell | NA | | | | |
| Alzheimer's_disease | Total_fatty_acids | | 6.27E-03 | 1.65E-02 | 7.24E-01 |
|  | Polyunsaturated_fatty_acids | | 7.86E-03 | 2.52E-02 | 7.71E-01 |
|  | Saturated_fatty_acids | | 1.70E-03 | 1.22E-02 | 8.96E-01 |
|  | Vitamin_D | | 2.76E-04 | 4.15E-04 | 5.74E-01 |
|  | Vitamin_E | | -3.13E-03 | 6.80E-03 | 6.77E-01 |
|  | Fresh_tomato_intake | | 5.22E-03 | 8.29E-03 | 5.73E-01 |
|  | Coffee_intake | | 3.30E-04 | 2.15E-03 | 8.85E-01 |
|  | Alcoholic_drinks_per_week | | 2.58E-03 | 1.97E-03 | 2.60E-01 |
|  | Lifetime_number_of_sexual_partners | | -2.11E-03 | 2.06E-03 | 3.64E-01 |
|  | Cigarettes_smoked_per_day | | -4.65E-03 | 1.11E-02 | 6.98E-01 |
|  | Fried_potatoes_intake | | 6.23E-03 | 7.84E-03 | 4.85E-01 |
| **Heterogeneity test** | | | | | |
| **Exposure** | **Outcome** | **Method** | **Q statistic** | **Q_df** | **Pheterogeneity** |
| Total_fatty_acids | Liver_iron_content | IVW | 137.749 | 61 | 7.29E-08 |
|  | Liver_iron_content | MR Egger | 136.004 | 60 | 7.90E-08 |
|  | Sex_hormone_binding_globulin_levels | IVW | 5702.741 | 61 | 0.00E+00 |
|  | Sex_hormone_binding_globulin_levels | MR Egger | 5689.308 | 60 | 0.00E+00 |
|  | Circulating_leptin_levels | IVW | 75.048 | 20 | 2.67E-08 |
|  | Circulating_leptin_levels | MR Egger | 72.901 | 19 | 3.01E-08 |
|  | Naive_CD4-CD8-_T_cell %T_cell | IVW | 74.338 | 55 | 4.22E-02 |
|  | Naive_CD4-CD8-_T_cell %T_cell | MR Egger | 74.054 | 54 | 3.64E-02 |
|  | Alzheimer's_disease | IVW | 50.669 | 59 | 7.72E-01 |
|  | Alzheimer's_disease | MR Egger | 49.916 | 58 | 7.66E-01 |
| Saturated_fatty_acids | Liver_iron_content | IVW | 111.359 | 51 | 2.19E-06 |
|  | Liver_iron_content | MR Egger | 104.494 | 50 | 1.01E-05 |
|  | Sex_hormone_binding_globulin_levels | IVW | 5248.918 | 51 | 0.00E+00 |
|  | Sex_hormone_binding_globulin_levels | MR Egger | 5181.235 | 50 | 0.00E+00 |
|  | Circulating_leptin_levels | IVW | 70.167 | 16 | 9.33E-09 |
|  | Circulating_leptin_levels | MR Egger | 69.927 | 15 | 4.60E-09 |
|  | Naive_CD4-CD8-_T_cell %T_cell | IVW | 71.070 | 49 | 2.13E-02 |
|  | Naive_CD4-CD8-_T_cell %T_cell | MR Egger | 70.976 | 48 | 1.72E-02 |
|  | Alzheimer's_disease | IVW | 52.469 | 49 | 3.41E-01 |
|  | Alzheimer's_disease | MR Egger | 52.128 | 48 | 3.17E-01 |
| Polyunsaturated_fatty_acids | Liver_iron_content | IVW | 121.638 | 63 | 1.32E-05 |
|  | Liver_iron_content | MR Egger | 121.369 | 62 | 9.95E-06 |
|  | Sex_hormone_binding_globulin_levels | IVW | 4163.758 | 63 | 0.00E+00 |
|  | Sex_hormone_binding_globulin_levels | MR Egger | 4144.340 | 62 | 0.00E+00 |
|  | Circulating_leptin_levels | IVW | 30.788 | 22 | 1.01E-01 |
|  | Circulating_leptin_levels | MR Egger | 30.735 | 21 | 7.82E-02 |
|  | Naive_CD4-CD8-_T_cell %T_cell | IVW | 62.865 | 57 | 2.76E-01 |
|  | Naive_CD4-CD8-_T_cell %T_cell | MR Egger | 62.810 | 56 | 2.48E-01 |
|  | Alzheimer's_disease | IVW | 415.997 | 56 | 1.90E-56 |
|  | Alzheimer's_disease | MR Egger | 394.964 | 55 | 6.44E-53 |
| Vitamin_D | NA | | | | |
| Vitamin_E | NA | | | | |
| Fresh_tomato_intake | NA | | | | |
| Coffee_intake | Liver_iron_content | IVW | 64.658 | 38 | 4.47E-03 |
|  | Liver_iron_content | MR Egger | 64.549 | 37 | 3.35E-03 |
|  | Sex_hormone_binding_globulin_levels | IVW | 1837.022 | 38 | 0.00E+00 |
|  | Sex_hormone_binding_globulin_levels | MR Egger | 1800.667 | 37 | 0.00E+00 |
|  | Circulating_leptin_levels | IVW | 96.748 | 8 | 1.97E-17 |
|  | Circulating_leptin_levels | MR Egger | 70.680 | 7 | 1.08E-12 |
|  | Naive_CD4-CD8-_T_cell %T_cell | IVW | 43.683 | 38 | 2.43E-01 |
|  | Naive_CD4-CD8-_T_cell %T_cell | MR Egger | 43.584 | 37 | 2.12E-01 |
|  | Alzheimer's_disease | IVW | 37.452 | 37 | 4.48E-01 |
|  | Alzheimer's_disease | MR Egger | 37.446 | 36 | 4.03E-01 |
| Alcoholic_drinks_per_week | Liver_iron_content | IVW | 54.699 | 32 | 7.47E-03 |
|  | Liver_iron_content | MR Egger | 53.865 | 31 | 6.65E-03 |
|  | Sex_hormone_binding_globulin_levels | IVW | 2530.832 | 33 | 0.00E+00 |
|  | Sex_hormone_binding_globulin_levels | MR Egger | 2490.081 | 32 | 0.00E+00 |
|  | Circulating_leptin_levels | IVW | 38.069 | 6 | 1.09E-06 |
|  | Circulating_leptin_levels | MR Egger | 37.464 | 5 | 4.84E-07 |
|  | Naive_CD4-CD8-_T_cell %T_cell | IVW | 42.982 | 33 | 1.14E-01 |
|  | Naive_CD4-CD8-_T_cell %T_cell | MR Egger | 42.967 | 32 | 9.33E-02 |
|  | Alzheimer's_disease | IVW | 38.239 | 33 | 2.44E-01 |
|  | Alzheimer's_disease | MR Egger | 38.181 | 32 | 2.09E-01 |
| Lifetime_number_of_sexual_partners | Liver_iron_content | IVW | 69.278 | 60 | 1.93E-01 |
|  | Liver_iron_content | MR Egger | 69.278 | 59 | 1.69E-01 |
|  | Sex_hormone_binding_globulin_levels | IVW | 334.576 | 60 | 9.20E-40 |
|  | Sex_hormone_binding_globulin_levels | MR Egger | 327.674 | 59 | 6.70E-39 |
|  | Circulating_leptin_levels | IVW | 6.487 | 6 | 3.71E-01 |
|  | Circulating_leptin_levels | MR Egger | 6.056 | 5 | 3.01E-01 |
|  | Naive_CD4-CD8-_T_cell %T_cell | IVW | 66.955 | 59 | 2.23E-01 |
|  | Naive_CD4-CD8-_T_cell %T_cell | MR Egger | 66.698 | 58 | 2.03E-01 |
|  | Alzheimer's_disease | IVW | 94.375 | 59 | 2.35E-03 |
|  | Alzheimer's_disease | MR Egger | 92.538 | 58 | 2.66E-03 |
| Cigarettes_smoked_per_day | Liver_iron_content | IVW | 66.570 | 21 | 1.23E-06 |
|  | Liver_iron_content | MR Egger | 63.346 | 20 | 2.13E-06 |
|  | Sex_hormone_binding_globulin_levels | IVW | 148.353 | 21 | 3.61E-21 |
|  | Sex_hormone_binding_globulin_levels | MR Egger | 128.667 | 20 | 6.93E-18 |
|  | Circulating_leptin_levels | IVW | 2.821 | 1 | 9.30E-02 |
|  | Circulating_leptin_levels | MR Egger | NA | | |
|  | Naive_CD4-CD8-_T_cell %T_cell | IVW | 22.057 | 21 | 3.96E-01 |
|  | Naive_CD4-CD8-_T_cell %T_cell | MR Egger | 17.689 | 20 | 6.08E-01 |
|  | Alzheimer's_disease | IVW | 27.204 | 21 | 1.64E-01 |
|  | Alzheimer's_disease | MR Egger | 26.631 | 20 | 1.46E-01 |
| Fried_potatoes_intake | NA | | | | |
| Liver_iron_content | Total_fatty_acids | IVW | 42.262 | 9 | 2.94E-06 |
|  | Total_fatty_acids | MR Egger | 39.035 | 8 | 4.84E-06 |
|  | Polyunsaturated_fatty_acids | IVW | 43.085 | 9 | 2.08E-06 |
|  | Polyunsaturated_fatty_acids | MR Egger | 37.955 | 8 | 7.67E-06 |
|  | Saturated_fatty_acids | IVW | 34.900 | 9 | 6.21E-05 |
|  | Saturated_fatty_acids | MR Egger | 33.202 | 8 | 5.66E-05 |
|  | Vitamin_D | IVW | 6.772 | 7 | 4.53E-01 |
|  | Vitamin_D | MR Egger | 6.729 | 6 | 3.47E-01 |
|  | Vitamin_E | IVW | 5.943 | 7 | 5.46E-01 |
|  | Vitamin_E | MR Egger | 5.711 | 6 | 4.56E-01 |
|  | Fresh_tomato_intake | IVW | 4.579 | 7 | 7.11E-01 |
|  | Fresh_tomato_intake | MR Egger | 3.920 | 6 | 6.88E-01 |
|  | Coffee_intake | IVW | 15.308 | 7 | 3.22E-02 |
|  | Coffee_intake | MR Egger | 15.303 | 6 | 1.80E-02 |
|  | Alcoholic_drinks_per_week | IVW | 6.538 | 6 | 3.66E-01 |
|  | Alcoholic_drinks_per_week | MR Egger | 6.039 | 5 | 3.02E-01 |
|  | Lifetime_number_of_sexual_partners | IVW | 7.237 | 7 | 4.05E-01 |
|  | Lifetime_number_of_sexual_partners | MR Egger | 5.316 | 6 | 5.04E-01 |
|  | Cigarettes_smoked_per_day | IVW | 3.295 | 6 | 7.71E-01 |
|  | Cigarettes_smoked_per_day | MR Egger | 1.425 | 5 | 9.22E-01 |
|  | Fried_potatoes_intake | IVW | 10.608 | 7 | 1.57E-01 |
|  | Fried_potatoes_intake | MR Egger | 10.114 | 6 | 1.20E-01 |
| Sex_hormone_binding_globulin_levels | Total_fatty_acids | IVW | 2938.246 | 385 | 0.00E+00 |
|  | Total_fatty_acids | MR Egger | 2932.293 | 384 | 0.00E+00 |
|  | Polyunsaturated_fatty_acids | IVW | 3146.882 | 385 | 0.00E+00 |
|  | Polyunsaturated_fatty_acids | MR Egger | 3103.506 | 384 | 0.00E+00 |
|  | Saturated_fatty_acids | IVW | 2664.341 | 385 | 0.00E+00 |
|  | Saturated_fatty_acids | MR Egger | 2661.196 | 384 | 0.00E+00 |
|  | Vitamin_D | IVW | 411.183 | 343 | 6.69E-03 |
|  | Vitamin_D | MR Egger | 407.929 | 342 | 8.20E-03 |
|  | Vitamin_E | IVW | 469.749 | 350 | 1.91E-05 |
|  | Vitamin_E | MR Egger | 465.207 | 349 | 3.01E-05 |
|  | Fresh_tomato_intake | IVW | 387.386 | 350 | 8.22E-02 |
|  | Fresh_tomato_intake | MR Egger | 381.637 | 349 | 1.11E-01 |
|  | Coffee_intake | IVW | 1190.086 | 350 | 4.89E-92 |
|  | Coffee_intake | MR Egger | 1181.608 | 349 | 5.32E-91 |
|  | Alcoholic_drinks_per_week | IVW | 1006.259 | 345 | 6.32E-66 |
|  | Alcoholic_drinks_per_week | MR Egger | 1003.044 | 344 | 1.07E-65 |
|  | Lifetime_number_of_sexual_partners | IVW | 697.727 | 350 | 2.52E-25 |
|  | Lifetime_number_of_sexual_partners | MR Egger | 688.877 | 349 | 1.64E-24 |
|  | Cigarettes_smoked_per_day | IVW | 629.694 | 345 | 6.53E-19 |
|  | Cigarettes_smoked_per_day | MR Egger | 625.009 | 344 | 1.41E-18 |
|  | Fried_potatoes_intake | IVW | 398.849 | 350 | 3.66E-02 |
|  | Fried_potatoes_intake | MR Egger | 398.819 | 349 | 3.38E-02 |
| Circulating_leptin_levels | Total_fatty_acids | IVW | 13.048 | 2 | 1.47E-03 |
|  | Total_fatty_acids | MR Egger | 13.015 | 1 | 3.09E-04 |
|  | Polyunsaturated_fatty_acids | IVW | 2.405 | 2 | 3.00E-01 |
|  | Polyunsaturated_fatty_acids | MR Egger | 2.101 | 1 | 1.47E-01 |
|  | Saturated_fatty_acids | IVW | 13.104 | 2 | 1.43E-03 |
|  | Saturated_fatty_acids | MR Egger | 13.015 | 1 | 3.09E-04 |
|  | Vitamin_D | IVW | 6.770 | 2 | 3.39E-02 |
|  | Vitamin_D | MR Egger | 6.197 | 1 | 1.28E-02 |
|  | Vitamin_E | IVW | 12.118 | 2 | 2.34E-03 |
|  | Vitamin_E | MR Egger | 11.894 | 1 | 5.63E-04 |
|  | Fresh_tomato_intake | IVW | 1.030 | 2 | 5.98E-01 |
|  | Fresh_tomato_intake | MR Egger | 0.990 | 1 | 3.20E-01 |
|  | Coffee_intake | IVW | 51.743 | 2 | 5.81E-12 |
|  | Coffee_intake | MR Egger | 25.273 | 1 | 4.98E-07 |
|  | Alcoholic_drinks_per_week | IVW | 28.566 | 2 | 6.26E-07 |
|  | Alcoholic_drinks_per_week | MR Egger | 21.903 | 1 | 2.87E-06 |
|  | Lifetime_number_of_sexual_partners | IVW | 19.870 | 2 | 4.84E-05 |
|  | Lifetime_number_of_sexual_partners | MR Egger | 15.496 | 1 | 8.27E-05 |
|  | Cigarettes_smoked_per_day | IVW | 0.769 | 2 | 6.81E-01 |
|  | Cigarettes_smoked_per_day | MR Egger | 0.433 | 1 | 5.11E-01 |
|  | Fried_potatoes_intake | IVW | 7.860 | 2 | 1.96E-02 |
|  | Fried_potatoes_intake | MR Egger | 0.095 | 1 | 7.58E-01 |
| Naive_CD4-CD8-_T_cell %T_cell | NA | | | | |
| Alzheimer's_disease | Total_fatty_acids | IVW | 63.090 | 5 | 2.79E-12 |
|  | Total_fatty_acids | MR Egger | 60.898 | 4 | 1.88E-12 |
|  | Polyunsaturated_fatty_acids | IVW | 150.791 | 5 | 9.06E-31 |
|  | Polyunsaturated_fatty_acids | MR Egger | 147.223 | 4 | 8.01E-31 |
|  | Saturated_fatty_acids | IVW | 33.692 | 5 | 2.74E-06 |
|  | Saturated_fatty_acids | MR Egger | 33.531 | 4 | 9.30E-07 |
|  | Vitamin_D | IVW | 1.260 | 3 | 7.39E-01 |
|  | Vitamin_D | MR Egger | 0.817 | 2 | 6.65E-01 |
|  | Vitamin_E | IVW | 4.635 | 4 | 3.27E-01 |
|  | Vitamin_E | MR Egger | 4.330 | 3 | 2.28E-01 |
|  | Fresh_tomato_intake | IVW | 2.931 | 4 | 5.69E-01 |
|  | Fresh_tomato_intake | MR Egger | 2.534 | 3 | 4.69E-01 |
|  | Coffee_intake | IVW | 6.715 | 5 | 2.43E-01 |
|  | Coffee_intake | MR Egger | 6.676 | 4 | 1.54E-01 |
|  | Alcoholic_drinks_per_week | IVW | 4.556 | 5 | 4.72E-01 |
|  | Alcoholic_drinks_per_week | MR Egger | 2.839 | 4 | 5.85E-01 |
|  | Lifetime_number_of_sexual_partners | IVW | 5.913 | 5 | 3.15E-01 |
|  | Lifetime_number_of_sexual_partners | MR Egger | 4.688 | 4 | 3.21E-01 |
|  | Cigarettes_smoked_per_day | IVW | 15.991 | 5 | 6.87E-03 |
|  | Cigarettes_smoked_per_day | MR Egger | 15.326 | 4 | 4.07E-03 |
|  | Fried_potatoes_intake | IVW | 5.490 | 4 | 2.41E-01 |
|  | Fried_potatoes_intake | MR Egger | 4.535 | 3 | 2.09E-01 |

Abbreviations: PCs=prostate cancers; TL=telomere length; IVW=inverse variance weighted; MR=Mendelian Randomization
